# Contribution of A1 to macrophage survival in cooperation with MCL-1 and BCL-X_L_ in a murine cell model of myeloid differentiation

**DOI:** 10.1038/s41419-024-07064-z

**Published:** 2024-09-16

**Authors:** Juliane Vier, Georg Häcker, Susanne Kirschnek

**Affiliations:** Institute of Medical Microbiology and Hygiene, Medical Center, University of Freiburg, Faculty of Medicine, Freiburg, Germany

**Keywords:** Apoptosis, Cell death and immune response, Immune cell death, Inflammation, Phagocytes

## Abstract

Myeloid cells are the first line of defence against pathogens. Mitochondrial apoptosis signalling is a crucial regulator of myeloid cell lifespan and modulates the function of myeloid cells. The anti-apoptotic protein BCL-2-family protein BCL2A1/A1/BFL-1 is strongly upregulated in inflammation in macrophages. We analysed the contribution of A1 to apoptosis regulation in a conditional system of in vitro differentiation of murine macrophages from immortalised progenitors. We disabled the expression of A1 by targeting all murine A1 isoforms in the genome. Specific inhibitors were used to inactivate other anti-apoptotic proteins. Macrophage progenitor survival mainly depended on the anti-apoptotic proteins MCL-1, BCL-X_L_ and A1 but not BCL-2. Deletion of A1 on its own had little effect on progenitor cell survival but was sensitised to cell death induction when BCL-X_L_ or MCL-1 was neutralised. In progenitors, A1 was required for survival in the presence of the inflammatory stimulus LPS. Differentiated macrophages were resistant to inhibition of single anti-apoptotic proteins, but A1 was required to protect macrophages against inhibition of either BCL-X_L_ or MCL-1; BCL-2 only had a minor role in these cells. Cell death by neutralisation of anti-apoptotic proteins completely depended on BAX with a small contribution of BAK only in progenitors in the presence of LPS. A1 and NOXA appeared to stabilise each other at the posttranscriptional level suggesting direct binding. Co-immunoprecipitation experiments showed the binding of A1 to NOXA and BIM. Interaction between A1 and Noxa may indirectly prevent neutralisation and destabilization of MCL-1. Our findings suggest a unique role for A1 as a modulator of survival in the macrophage lineage in concert with MCL-1 and BCL-X_L_, especially in a pro-inflammatory environment.

## Introduction

Mitochondrial apoptosis is regulated by the BCL-2 protein family, which contains anti-apoptotic and pro-apoptotic proteins classified according to their structure and function. Five anti-apoptotic BCL-2-like proteins exist in mammals (BCL-2, BCL-X_L_, MCL-1, BCL-W and A1/BFL-1). All can block apoptosis by binding to and inhibiting the pro-apoptotic family members, but they differ strongly with respect to a number of features such as expression, binding affinity to pro-apoptotic members and turnover.

The roles of MCL-1, BCL-2 and BCL-X_L_ in the hematopoietic compartment are well-characterised [1, 2]. The roles of BCL-w and A1/BFL-1, however, are less clear. BCL-w was originally described to protect lymphoid and myeloid cells against apoptosis induced by cytokine withdrawal or γ-irradiation when overexpressed [3]. It is required for spermatogenesis and is expressed in hematopoietic cells but is dispensable for the development of hematopoietic compartments [4, 5]. While overexpressed in certain B-cell lymphoma lines, BCL-w seems to have a minor role in lymphoma development [6, 7]. It was shown to be redundant for granulocyte survival [8], however, its role in other myeloid cells seems still not clear. A1/BFL-1 is far less well investigated as well. This has been due to low levels of expression or low sensitivity of available antibodies, the lack of specific inhibitors or perhaps a more limited function of the protein. One aspect in mice that has also complicated the study of A1 is the presence of four individual genes (A1a-d) (three functional: A1c is a non-functional fragment) with presumably similar functions [9].

The isoform A1a was originally isolated from mouse bone marrow as a GM-CSF inducible, hematopoietic tissue-specific gene [10, 11]. The human counterpart BFL-1 was first isolated from human fetal liver [12]. A1 is mainly expressed in the hematopoietic compartment and is strongly induced by a number of pro-inflammatory stimuli [13]. Deletion of A1a resulted in a reduced lifespan of neutrophils ex vivo [14]. An inducible shRNA knockdown approach targeting all isoforms revealed a role of A1 in early T-cell differentiation, B-cell homoeostasis, IgE-mediated mast cell-driven anaphylaxis, and granulocyte survival [15, 16]. An A1a,b,d-deficient mouse (lacking all functional isoforms) had only minor defects: a minor decrease in conventional dendritic cell (cDC) numbers and impaired cDC survival were noted [17], as were minor T-cell defects [18]. Loss of A1 increased the sensitivity of cDCs to BCL-2 neutralisation [19]. Lack of A1 also accelerated neutrophil spontaneous and FAS-ligand-induced apoptosis [20]. A recently published study found that A1 negatively regulated BAX/BAK-dependent apoptosis and subsequent inflammasome- and caspase-8-mediated maturation of the inflammatory cytokine IL-1β following LPS treatment in bone marrow-derived mouse macrophages and monocytes [21].

A number of reports found upregulation of A1 in various tumours, especially of hematopoietic origin, correlating with resistance to chemotherapy; this was shown for chronic lymphocytic leukaemia [22], B-cell lymphoma [23, 24] and acute promyelocytic leukaemia [25, 26]. In melanoma, high levels of BFL-1 were associated with chemoresistance [27, 28]. Deletion of A1 sensitised melanoma cells to BCL-2-protein targeting drugs [29].

BFL-1 was overexpressed in B-cells from lupus patients [30] and could serve as a urinary biomarker in lupus nephritis [31]. A1 was characterised as a potential prognostic and diagnostic biomarker for human sepsis [32, 33], although in vivo infection models in the mouse did not show any impact of A1 deletion on immune cell survival [34].

We use a model of differentiation of myeloid cells from mouse progenitors to assess the role of BCL-2-family proteins at various stages and cell lineages. The model is based on the regulated expression of Hoxb8 and permits the generation of progenitor cells committed to various lineages, especially neutrophils and macrophages/monocytes [35, 36]. Using this model, we have previously investigated the role of BCL-2-family proteins in neutrophil progenitors and mature neutrophils [37] and have assessed the role of A1 in cells from the neutrophil lineage [38]. We here analysed the role of A1 in macrophage progenitors and differentiated macrophages in the Hoxb8-model.

We tested the dynamics of cell death induction, BCL-2 family protein expression and interaction profiles to identify differential roles of A1 and the main known regulators of macrophage cell death_,_ in survival regulation in the monocyte/macrophage lineage.

## Results

### Contribution of A1 to survival in a murine macrophage differentiation model

In macrophages differentiated from mouse bone marrow in vitro, A1 is induced by LPS and then contributes to cell survival if other BCL-2-proteins are inhibited [21]. Macrophages differentiate from myeloid bone marrow progenitors, and the composition of BCL-2-family members and their individual contributions to cell survival likely change during differentiation. We used a model of committed mouse macrophage progenitors to test the role of A1 in progenitors and in mature macrophages, at resting state or when stimulated with LPS.

In this model, conditionally active oestrogen receptor-regulated Hoxb8 (ER-Hoxb8) drives the expansion of committed macrophage progenitors from mouse bone marrow in presence of GM-CSF. When Hoxb8 is turned off (oestrogen withdrawal), the cells differentiate into macrophages [35, 36]. We first derived and tested GM-CSF-dependent progenitors from wt mice.

Potent and specific chemical inhibitors against MCL-1, BCL-2 or BCL-X_L_ exist [39, 40], but no specific small molecule inhibitor of A1 has been introduced. Complete deletion of murine A1 is complicated by the presence of three functional genes. We used lentiviral CRISPR/Cas9 to target all isoforms simultaneously using one single gRNA. We devised two different sgRNAs, each targeting all isoforms, to establish A1-deficient GM-SCF Hoxb8 macrophage progenitor cells, with A1g7 matching the sequence of all isoforms (A1a,b,c,d), and A1g12 with one nucleotide mismatch in A1b. Efficient loss of A1 protein in progenitors was confirmed for A1g7 by Immunoblotting (Fig. S1). Cells expressing A1g12 showed a remaining faint band, suggesting that the genomic manipulation was largely successful, but A1 loss was not complete, possibly due to insufficient targeting of A1b (we will refer to these A1-targeted cells as A1-KO).

**Fig. 1:**
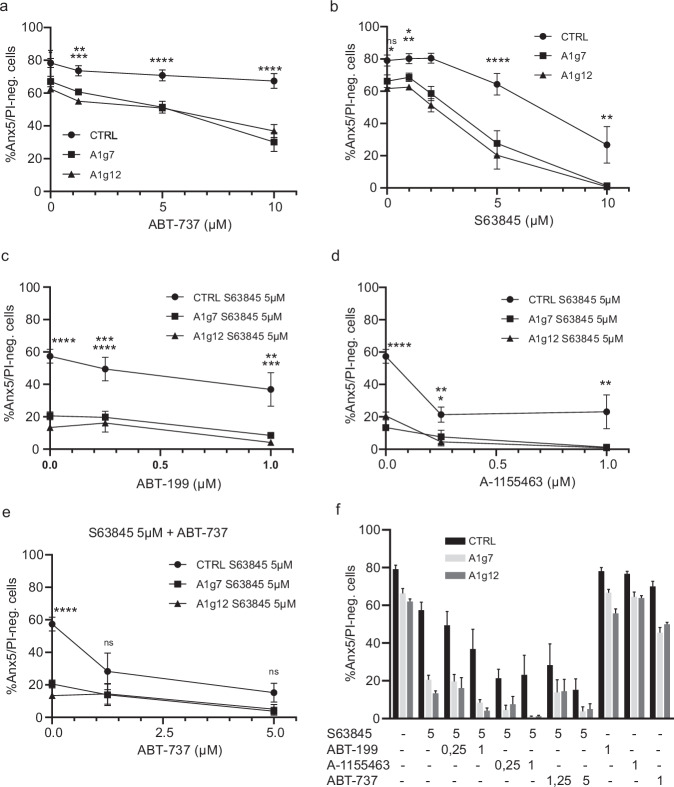
Targeting anti-apoptotic proteins in macrophage progenitors reveals an essential role of MCL-1 in cooperation with A1 and BCL-X_L_ for survival in steady state. A1 was knocked out in Hoxb8 GM-SCF macrophage progenitors by lentiviral CRISPR/Cas9 using two different sgRNAs (A1g7 and A1g12) targeting all isoforms (A1g7) or A1a, c and d (A1g12). An irrelevant sgRNA sequence directed against EGFP served as control (CTRL). **a**–**d** Hoxb8 macrophage progenitors were treated for 24 h with the following inhibitors targeting anti-apoptotic BCL-2-family proteins: ABT-737 (**a**, specific for BCL-X_L_ and BCL-2), S63845 (**b**, specific for MCL-1), ABT-199 (**c**, specific for BCL-2) or A-1155463 (**d**, specific for BCL-X_L_) at the concentrations (µM) indicated. **e**, **f** Hoxb8 macrophage progenitors were treated as above with single or combined inhibitors as indicated. DMSO served as solvent control. Cell death was assessed by AnnexinV/PI staining and flow cytometry (FACS Calibur). Shown are the percentage of live cells (AnnexinV and PI-negative). Data are means/SEM of 3–7 independent experiments. Statistical analysis was done by 2-way ANOVA. *p < 0.05; **p < 0.01; ***p < 0.001; ****p < 0.0001; ns, non-significant.

A1 deletion had little effect on macrophage progenitors in terms of cell death under homoeostatic conditions. There was a small but significant increase in background cell death in both A1-targeted progenitor lines A1g7 and A1g12 compared to control cells expressing an irrelevant EGFP-targeting gRNA (CTRL) (Fig. 1a, b). We then treated A1-KO or CTRL progenitors with specific inhibitors targeting MCL-1 (S63845) or BCL-2/BCL-XL (ABT-737) and analysed cell death by AnnexinV/PI staining (Fig. 1, after 24 h) and/or staining against active caspase-3 (Fig. S3). Inhibition of BCL-2/BCL-X_L_ had very minor effects on the viability of CTRL progenitors even at high ABT-737 concentrations but led to substantially decreased survival in both A1-KO lines (Fig. 1a). MCL-1 inhibition strongly impaired survival in CTRL. Additional A1 deletion blocked survival near-completely at the highest MCL-1-inhibitor concentration (Fig. 1b). Specific inhibition of either BCL-2 or BCL-X_L_ alone did not affect progenitor survival in CTRL or A1-KO lines (Fig. 1f, Fig. S3). Inhibition of MCL-1 combined with BCL-X_L_ inhibition, but less so with BCL-2 inhibition, had a synergistic effect in killing CTRL cells, similar to combined treatment with MCL-1 inhibitor and ABT-737. BCL-X_L_- combined with MCL-1 inhibition and A1 deficiency blocked cell survival completely (Fig. 1c–f). Progenitor survival thus was mainly dependent on MCL-1, BCL-X_L_ and A1, but not BCL-2, with MCL-1 being the most important. A1, however, made a very substantial contribution to keep the cells alive when other anti-apoptotic proteins were neutralised.

### Effect of differentiation on myeloid cell dependency on anti-apoptotic Bcl-2-family proteins

**Fig. 2:**
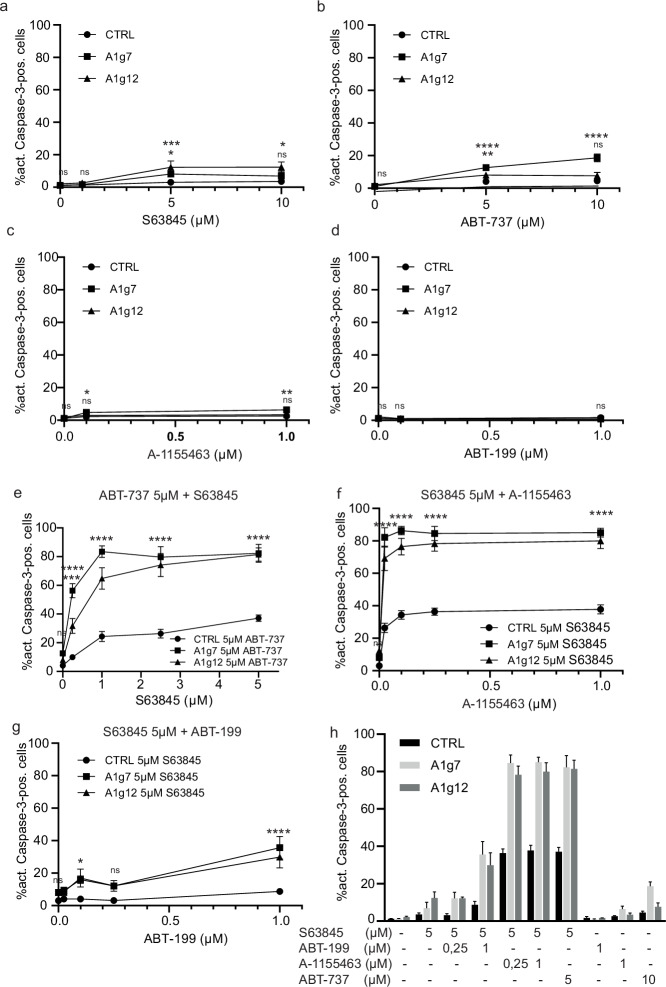
Differentiation confers resistance to inhibition of single anti-apoptotic proteins, which can be overcome by combined neutralisation of A1, MCL-1 and BCL-X_L_. A1-KO or CTRL GM-CSF Hoxb8 cells (A1g7, A1g12 or CTRL) were differentiated into macrophages for 7 days by oestrogen withdrawal. **a**–**d** Differentiated macrophages were treated for 4 h with the following inhibitors targeting anti-apoptotic BCL-2-family proteins: ABT-737 (**a**, specific for BCL-X_L_ and BCL-2), S63845 (**b**, specific for MCL-1), ABT-199 (**c**, specific for BCL-2) or A-1155463 (**d**, specific for BCL-X_L_) at the concentrations (µM) indicated. **e**–**h** Differentiated macrophages were treated as above with single or combined inhibitors as indicated. DMSO served as solvent control. Cell death was assessed by active-Caspase-3 staining and flow cytometry (FACS Calibur). Data are means/SEM of 3–7 independent experiments. Statistical analysis was done by 2-way ANOVA. *p < 0.05; **p < 0.01; ***p < 0.001; ****p < 0.0001; ns, non-significant.

Cell differentiation may have effects on apoptosis sensitivity. In order to investigate the effect of the differentiation status on the role of A1, we differentiated the progenitors into macrophages by oestrogen withdrawal for 7 days. A1-KO cells differentiated normally, with typical changes in morphology and surface marker expression characteristic for monocyte-derived macrophages (e.g., CD11b, F4/80, CD64 upregulation, c-Kit downregulation) (Fig. S2a, b). Functional analysis of phagocytosis and reactive oxygen species (ROS) production in differentiated macrophages did not reveal differences between genotypes. Both CTRL and A1-KO cells effectively phagocytosed *E. coli* ‘bioparticles’ and generated ROS upon PMA stimulation (Fig. S2c, d). Macrophages were treated with various doses of single or combined inhibitors of MCL-1, BCL-2 or BCL-X_L_, and cell death was analysed by monitoring active caspase-3 (Fig. 2) or loss of cell membrane integrity using a live-dead stain (Fig. S4a–h).

In contrast to progenitors, differentiated CTRL cells were resistant to all single inhibitor treatments, even at high doses. A1 deficiency on its own did not impair survival (Fig. 2a–d), but had weak pro-apoptotic effects in combination with single inhibitors against MCL-1 or BCL-2/BCL-X_L_ (Fig. 2a, b). Simultaneous MCL-1 inhibition and ABT-737 treatment strongly induced caspase-3 activation in combination with A1 deficiency, but only mildly increased cell death in CTRL (Fig. 2e). In order to test for the relevant protein targeted by ABT-737, we combined MCL-1 inhibition with increasing concentrations of BCL-X_L_- or BCL-2-specific inhibitor. In A1-deficient cells, low doses of BCL-X_L_ inhibitor in combination with MCL-1 inhibition induced strong caspase-3 activation, in contrast to CTRL cells (Fig. 2f). Combined BCL-2 and MCL-1 inhibition did not induce apoptosis in CTRL macrophages. Additional A1 deficiency slightly sensitised to cell death at high ABT-199 concentrations (Fig. 2g, h). Live-dead staining, including AnnexinV/PI staining, appeared clearly less sensitive than anti-active caspase-3 staining for the detection of mitochondrial apoptosis in differentiated cells (a direct comparison is shown in Fig. S4i, j), therefore staining against active caspase-3 was performed in all further experiments with differentiated macrophages, and was complemented by annexinV/PI staining for some experiments.

Taken together, differentiation confers strong resistance against the neutralisation of single anti-apoptotic proteins. This resistance can be overcome by concomitant neutralisation of additional anti-apoptotic proteins, where A1 clearly plays a role in keeping the cells alive.

### A1 is important for survival of macrophage progenitors under pro-inflammatory LPS stimulation

**Fig. 3:**
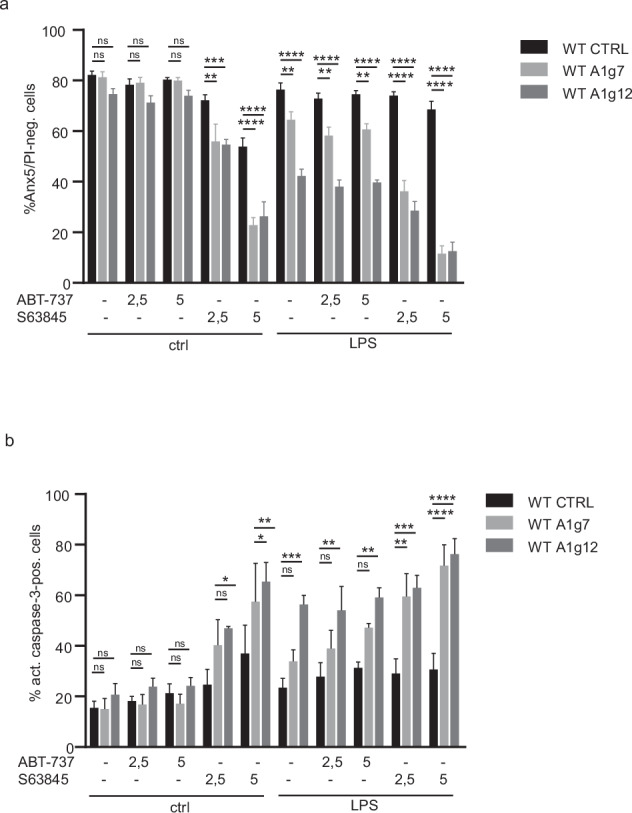
Pro-inflammatory stimulation by LPS sensitises progenitors to cell death in the absence of A1 but is able to protect when A1 is present. **a**, **b** A1-KO and WT CTRL GM-CSF progenitors were co-treated with LPS (1 µg/ml) and inhibitors against BCL-X_L_/BCL-2 (ABT-737) or MCL-1 (S63845) at the concentrations (µM) indicated. DMSO served as solvent control. Viability and apoptotic cell death were assessed after 4 h by AnnexinV/PI staining (**a**, shown are the percentage of live, AnnexinV and PI-negative cells) and staining against active caspase-3 (**b**) followed by flow cytometry analysis (FACS Calibur). Data are means/SEM of 3–7 independent experiments. Statistical analysis was done by 2-way ANOVA. *p < 0.05; **p < 0.01; ***p < 0.001; ****p < 0.0001; ns, non-significant.

A1 is an NFκB-inducible gene highly regulated in inflammation [41]. We therefore analysed the effect of inflammatory conditions on the role of A1 in apoptosis regulation of progenitors and differentiated macrophages. We used LPS as a well-characterised stimulus and simultaneously co-treated progenitors with different doses of MCL-1- or BCL-2/BCL-X_L_ inhibitors. Survival of CTRL cells was not altered by LPS stimulation alone, but A1 deficiency significantly sensitised progenitors to apoptosis upon LPS treatment (Fig. 3a, b). In CTRL cells, concomitant LPS stimulation reduced the pro-apoptotic effect of MCL-1 inhibition, but when A1 was absent, LPS, in contrast, enhanced this effect (Fig. 3a, b), indicating that the protective effect of LPS required A1. Neutralisation of BCL-2/BCL-X_L_ did not further compromise the viability of A1-deficient progenitors during LPS treatment. LPS thus conferred partial protection against MCL-1 inhibition in CTRL cells. This effect depended on A1. In progenitors, A1 deficiency turned LPS into a pro-apoptotic stimulus, even in the presence of functional MCL-1.

**Fig. 4:**
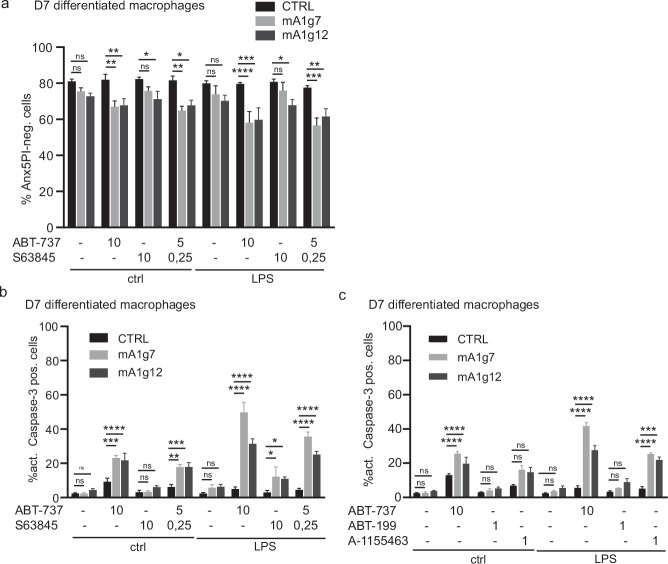
A1 deficiency promotes apoptosis in LPS-stimulated macrophages when MCL-1 or BCL-X_L_ is neutralised. **a**–**c** Day 7 differentiated Hoxb8 macrophages were co-treated with LPS and single or combined inhibitors against BCL-X_L_/BCL-2 (ABT-737), MCL-1 (S63845), BCL-2 (ABT-199) or BCL-X_L_ (A-1155463) at the concentrations (µM) indicated for 4 h. DMSO served as solvent control. Viability and apoptotic cell death were assessed by AnnexinV/PI staining (**a**) and staining against active caspase-3 (**b**, **c**), followed by flow cytometry (FACS Calibur). Data are means/SEM of 3 independent experiments. Statistical analysis was done by 2-way ANOVA. *p < 0.05; **p < 0.01; ***p < 0.001; ****p < 0.0001; ns, non-significant.

Although inflammatory conditions may occur during macrophage differentiation, contact with bacterial molecules such as LPS is probably more common in differentiated macrophages during infection. In differentiated macrophages, LPS stimulation alone did not compromise the survival of A1-deficient cells, in contrast to progenitors. However, A1 deficiency strongly sensitised to apoptosis under BCL-X_L_/BCL-2 inhibition and concomitant LPS stimulation (Fig. 4a–c). MCL-1 inhibition in the presence of LPS had a weaker pro-apoptotic effect in A1-deficient macrophages than in progenitors, contrary to BCL-X_L_/BCL-2 inhibition (Fig. 4a, b). Similar to progenitors, BCL-X_L_ was more important than BCL-2 for survival under LPS stimulation (Fig. 4c). Differentiation thus favoured general resistance to MCL-1 inhibition, but retained sensitivity to BCL-X_L_/BCL-2 neutralisation in A1-deficient cells especially under pro-inflammatory stimulation.

### Differential contributions of BAX and BAK to cell death regulation

**Fig. 5:**
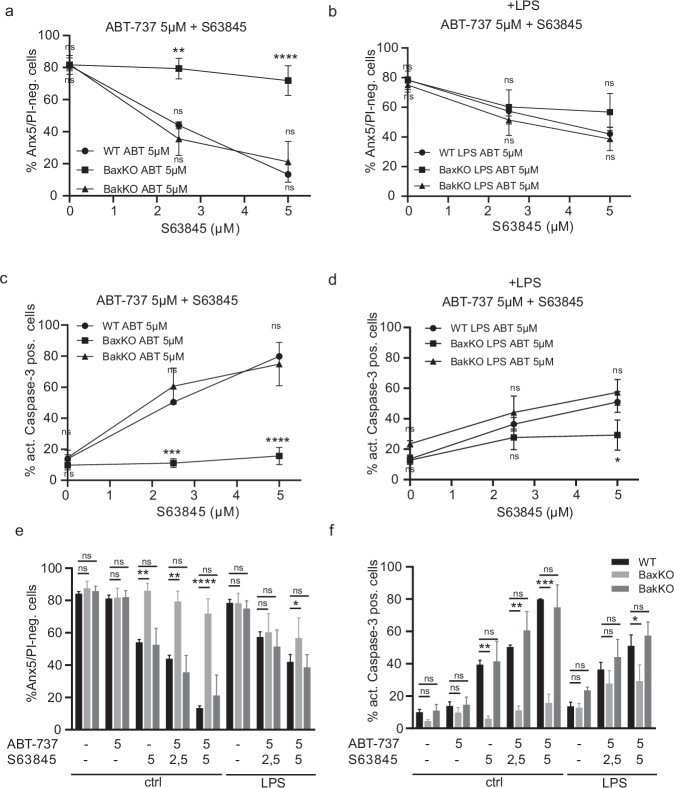
Role of BAX versus BAK for progenitor cell death upon neutralisation of anti-apoptotic proteins in steady state and under pro-inflammatory conditions. **a**–**f** WT, BAX-KO or BAK-KO progenitors were co-treated with LPS and combined inhibitors at the concentrations (µM) indicated. Viability and apoptotic cell death were assessed after 4 h by AnnexinV/PI staining (**a**, **b**, **e**) or staining against active caspase-3 (**c**, **d**, **f**) followed by flow cytometry (FACS Calibur). Data are means/SEM of 3 independent experiments. Statistical analysis was done by 2-way ANOVA. *p < 0.05; **p < 0.01; ***p < 0.001; ****p < 0.0001; ns, non-significant.

BAX and BAK are the effectors of cytochrome c release and mitochondrial apoptosis. Both proteins can be regulated by direct activation via BH3-only proteins and by neutralisation of anti-apoptotic BCL-2 family members. BAX and BAK are differentially regulated in terms of localisation within the cell (or retrotranslocation from mitochondria to cytosol) and binding profiles to BCL-2 family members. BCL-2 has been reported to sequester BAX but not BAK, whereas BAK may be restrained by MCL-1 and BCL-X_L_ [42]. In order to determine the respective contributions of each protein to apoptosis regulation in the macrophage differentiation model, we tested BAX or BAK single-deficient progenitors for sensitivity to simultaneous treatment with 5 µM ABT-737 and increasing concentrations of MCL-1 inhibitor. Whereas BAK-deficient cells appeared as sensitive as wt, BAX-deficient cells were completely resistant to S63845/ABT-737 treatment (Fig. 5a, c). Strikingly, the situation was different under inflammatory conditions: LPS co-treatment conferred partial protection against inhibitor treatment in BAK-deficient and wt cells, but slightly sensitised BAX-deficient cells to death. This suggests a certain role for BAK under inflammatory conditions, even if BAX-deficient cells were still the most protected (Fig. 5a–f).

**Fig. 6:**
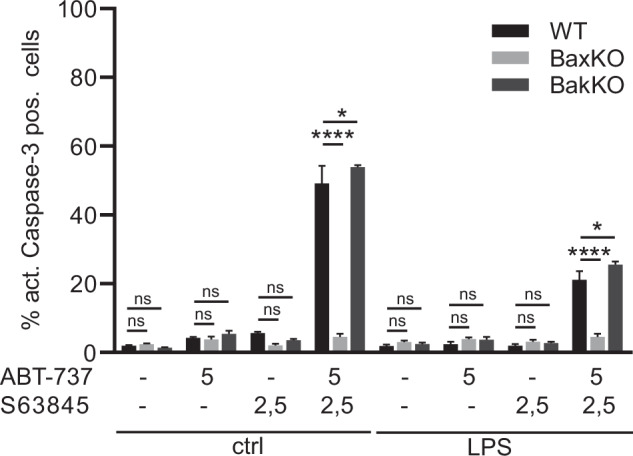
Role of BAX versus BAK in differentiated macrophages in the presence and absence of pro-inflammatory LPS stimulation. WT, BAX-KO or BAK-KO progenitors were co-treated with LPS and combined inhibitors at the concentrations (µM) indicated for 4 h. Apoptotic cell death was assessed by staining against active caspase followed by flow cytometry (FACS Calibur). Data are means/SEM of 3 independent experiments. Statistical analysis was done by 2-way ANOVA. *p < 0.05; **p < 0.01; ***p < 0.001; ****p < 0.0001; ns, non-significant.

In differentiated cells, as in progenitors, BAX deficiency resulted in complete protection against combined neutralisation of MCL-1, BCL-X_L_ and BCL-2. BAK deficiency had no protective effect (Fig. 6). LPS stimulation partially protected against inhibition of pro-survival proteins in both wt and BAK-deficient macrophages.

These results indicate that at resting state the function of anti-apoptotic BCL-2-family proteins is exclusively either the prevention of activation or the inhibition of BAX. Under pro-inflammatory stimulation, however, BAK apparently gained some importance in progenitors but not in differentiated macrophages. This indicates that the contribution of BAK to LPS-associated cell death is completely lost upon differentiation.

### Dynamics of Bcl2-family protein expression during differentiation

We used the macrophage differentiation model to monitor the expression of BCL-2 family members during the differentiation process. We observed an early induction of A1 and MCL-1 starting on the first day of differentiation (Fig. S5). BCL-2 and BCL-X_L_ were induced around d5. BCL-w showed no obvious change. The induction of anti-apoptotic proteins correlated with increased expression of the pro-apoptotic members BIM, NOXA, PUMA, BID, BAX and BAK. Although protein activity cannot simply be judged by protein expression levels, the increased expression of BCL-2-family members upon differentiation could, in part, explain the changes in apoptosis sensitivity seen in progenitors versus differentiated macrophages, with strong protection against single inhibition of anti-apoptotic proteins in differentiated macrophages but not in progenitors.

### Deficiency in A1 affects protein levels of other BCL-2-family proteins

**Fig. 7:**
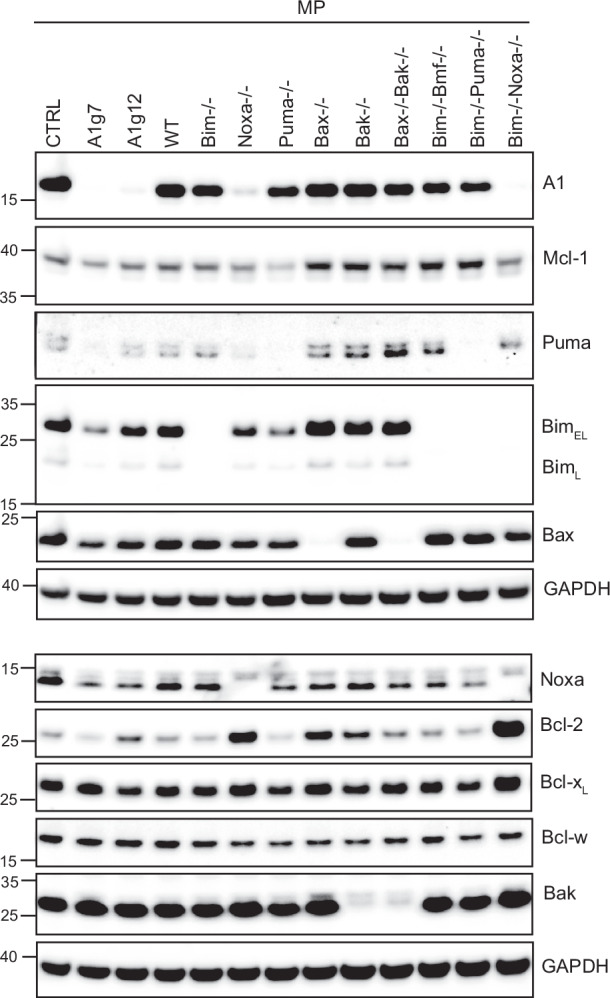
Analysis of protein levels of BCL-2-family proteins in progenitors deficient in A1 or in various pro-apoptotic BCL2-family proteins. WT progenitors or progenitors of the indicated genotypes were directly lysed in Bolt sample buffer, and samples were boiled at 70 °C for 10 min. Equal cell equivalents were separated by SDS-PAGE, transferred onto nitrocellulose membranes and probed with antibodies against various BCL-2 family proteins as indicated. GAPDH served as loading control. The asterisk denotes unspecific bands. Blots are representative of at least 2 experiments.

The absence or presence of distinct BCL-2 family members and interactions within the family may alter the protein stability of other members. A1 protein has a very short half-life, which may be an effect of strong regulation at the transcriptional, but also at the protein level [43]. We therefore investigated the impact of single/double deficiency of distinct pro-apoptotic proteins on A1 protein levels in progenitors, as well as the effects of A1 deficiency on levels of other proteins. Deficiency in NOXA, alone or combined with lack of BIM, strongly decreased A1 levels (Fig. 7). In contrast, combined BIM/PUMA or BIM/BMF deficiency did not substantially alter A1 expression. A1 deficiency, in turn, correlated with a prominent decrease in protein levels of NOXA, BIM and PUMA. Small changes in BAX and BCL-2, but not BAK or BCL-X_L_ levels, were also seen. These data suggest that A1 and NOXA may stabilise each other, possibly by direct binding. The results also suggest that BAX, but not BAK, may play a stabilising role in A1 turnover.

LPS stimulation strongly induced A1 and NOXA protein expression in CTRL cells in both progenitors and differentiated macrophages (Fig. S6). Hardly any induction of NOXA in A1-deficient cells was seen: whatever the mechanism of A1-dependent NOXA regulation, it therefore extended to the situation of pro-inflammatory stimulation. No clear changes in BIM, BAX, BAK or BCL-X_L_ levels were apparent. NOXA and A1 expression were thus strongly dependent on each other, suggesting that either direct interaction between the proteins or indirect effects may co-stabilise both proteins together.

To determine whether transcriptional alterations of A1 and NOXA expression could play a role in mutual regulation, we analysed A1 and NOXA mRNA levels of progenitors in LPS-stimulated samples. We found A1 mRNA upregulated in both CTRL and NOXA-deficient cells upon LPS stimulation, but NOXA-deficient cells showed significantly reduced A1 mRNA levels compared to CTRL cells in resting and stimulated samples (Fig. S7a). In A1-KO progenitors, NOXA mRNA levels were comparable to CTRL at resting state and slightly upregulated upon LPS stimulation, similar to CTRL progenitors (Fig. S7a). This indicates that NOXA can contribute to the regulation of A1 at the mRNA level, but not vice versa.

To test the role of the proteasome in the mutual stabilisation of A1 and NOXA, we treated LPS-stimulated CTRL or A1-KO cells with the proteasome inhibitor MG-132 for the last hour of stimulation and monitored A1 and NOXA protein levels. Proteasome inhibition stabilised NOXA in resting CTRL and A1-KO cells comparably (Fig. S7b). This indicated that proteasomal degradation was responsible for the decreased stability of NOXA in resting A1-KO progenitors. In LPS-stimulated CTRL cells, NOXA protein was increased and further accumulated under proteasome inhibition. When A1 was absent, however, LPS had hardly any effect on NOXA protein expression (Fig. S7b). MCL-1 levels in resting cells were not affected by lack of A1, and MCL-1-protein slightly increased upon proteasome inhibition, comparable in CTRL and A1-KO cells. However, MCL-1 protein accumulated in LPS/MG-132-treated CTRL cells, but not when A1 was absent. The results suggest that A1 contributes to stabilisation of NOXA-expression in LPS-treated progenitors at a posttranscriptional level, possibly guided by direct or indirect interactions within the BCL-2 family, where A1 may have indirect effects on MCL-1 stability.

### Interaction of A1 and MCL-1 with BCL-2-family members

**Fig. 8:**
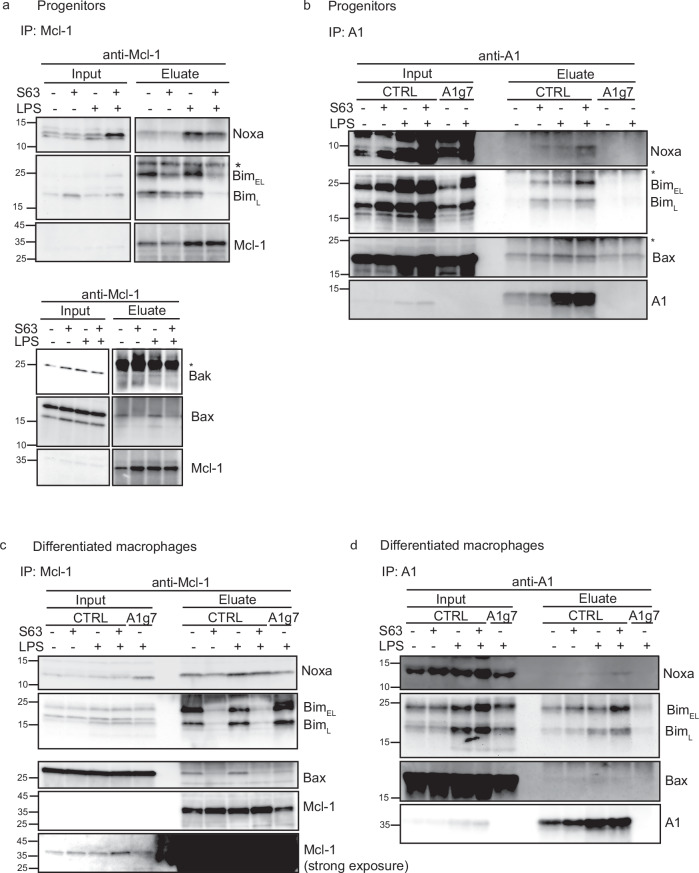
Interaction of MCL-1 and A1 with pro-apoptotic BCL-2 family proteins in macrophage progenitors. **a**, **b** Immunoprecipitation of A1 or MCL-1 and probing for interacting proteins in progenitors. WT CTRL (**a**, **b**) or A1-deficient A1g7 (**b**) macrophage progenitors were treated with the MCL-1 inhibitor S63845 (5 µM) in the presence or absence of LPS (1 µg/ml) for 18 h. QVD-OPH (20 µM) was added to all samples. Cells were lysed in 1% CHAPS lysis buffer and MCL-1 (**a**) or A1 (**b**) were precipitated using specific antibodies and Protein G agarose. Samples were eluted in 2x Laemmli buffer, boiled at 95 °C and separated by SDS-PAGE. Membranes were probed for co-precipitated proteins as indicated. Input was loaded as equivalents of 1/45 of the loaded IP precipitate, respectively. Iso, isotype control of pooled samples. The asterisk denotes unspecific bands. Data are representative of at least 3 experiments. **c**, **d** Immunoprecipitation of A1 or MCL-1 and probing for interacting proteins in differentiated macrophages. WT CTRL or A1-deficient A1g7 Hoxb8 cells were differentiated for 7 days by oestrogen withdrawal and then treated with the MCL-1 inhibitor S63845 (10 µM) in the presence or absence of LPS (1 µg/ml) for 6 h. Cells were lysed in CHAPS lysis buffer and immunoprecipitated with antibodies against Mcl-1 (**c**) or A1 (**d**). For Mcl-1IP, a flowthrough of the A1-IP was used. Precipitates were eluted in 2x Laemmli buffer by boiling at 95 °C for 5 min, separated by SDS-PAGE, transferred onto PVDF membranes and probed for co-precipitated proteins as indicated. Input samples were loaded as equivalents of 1/35 of the loaded IP precipitate. A1-KO cells served as specificity control for A1-IP. Data shown are representative of two independent experiments.

A1 and NOXA appear to regulate each other, and BAX but not BAK plays a role in apoptosis sensitivity upon loss of A1, particularly when MCL-1 is inhibited. We therefore tested complex formation between MCL-1 or A1 and other BCL-2-family members. MCL-1 readily co-precipitated BIM, NOXA, BAX and BAK from extracts of untreated progenitors (Fig. 8a). Strikingly, more NOXA was co-precipitated in LPS-stimulated versus untreated samples. As expected, Mcl-1 inhibition strongly reduced the binding of BAX and BAK to MCL-1.

When A1 was immunoprecipitated in untreated progenitors, BIM and BAX could be co-precipitated (Fig. 8b), although signals of co-precipitating proteins were weak and some unspecific binding of BAX was seen in A1-KO control samples. NOXA was hardly detectable. Upon MCL-1 inhibition, BIM and NOXA-binding to A1 was enhanced. LPS stimulation also increased the interaction of A1 with NOXA, BIM and BAX (Fig. 8b). No interaction of BAK with A1 could be detected (not shown). Differentiated macrophages showed similar interaction profiles of A1 and Mcl-1 with Noxa, Bim or Bax, as progenitors (Fig. 8c, d). The data thus indicate a shift of BIM from MCL-1 to A1 when MCL-1 is neutralised, and a prominent shift of BIM and NOXA to A1 under concurrent LPS stimulation in both progenitors and differentiated macrophages.

## Discussion

The understanding of the regulation of mitochondrial apoptosis in myeloid cells, especially in the macrophage lineage, is still limited. A1-function is little explored but appears to play a role in myeloid cells. We here identify a pro-survival role of A1, in cooperation with MCL-1 and BCL-X_L_ in macrophage precursor cells. Differentiation substantially altered the roles of the anti-apoptotic proteins, and A1 was particularly relevant in inflammatory conditions in progenitors. In differentiated cells, when BCL-X_L_ was neutralised, A1 was similarly important as MCL-1. Mutual stabilisation of A1 and NOXA at the posttranscriptional level suggests direct interaction. In resting cells, only BAX could kill, while in inflammatory conditions, BAK acquired some importance.

Anti-apoptotic BCL-2-family proteins inhibit cell death by binding to BH3-only proteins and BAX/BAK [44]. Much of what we have learned about these interactions has been derived from in vitro interaction studies of peptides/proteins or the use of BH3-mimetics [45]. It has become clear that MCL-1 has a binding profile different from BCL-2/BCL-X_L_/BCL-w [46], and neutralising MCL-1 kills many cancer cells efficiently [39]. A1 has an interaction pattern with BH3-peptides that is similar to MCL-1 [46]. This suggests that A1 and MCL-1 may be able to replace each other. Remarkably, while efficiently killing many cancer cells, MCL-1 inhibition is well tolerated by mice [39], indicating that non-transformed cells are naturally resistant to MCL-1 inhibition; cardiac damage of MCL-1 inhibitors is probably due to specific blockade of non-anti-apoptotic effects of MCL-1 [47]. Targeting of A1 in mice had a relatively subtle effect but this is also consistent with the view of a functional overlap of A1 and MCL-1-function.

Focusing on macrophage progenitors and macrophages, we found a profound effect of A1 deletion when other BCL-2 family members were inhibited. In the progenitor cells of our model, this effect was particularly pronounced for MCL-1 inhibition, suggesting that both proteins indeed have similar molecular functions. During differentiation, BCL-X_L_ also assumed an additional protective role and A1-deficient macrophages were efficiently killed by inhibition of MCL-1 and BCL-X_L_. Both MCL-1 and BCL-X_L_ were strongly upregulated during differentiation.

BAX and BAK are considered redundant in many situations. We found that in macrophage progenitors, apoptosis was exclusively implemented by BAX, and BAX deficiency provided near-complete protection against BH3-mimetics. This may simply reflect expression: upregulation during differentiation was much for pronounced for BAK. In any case, this much greater reliance on BAX in this cell type is interesting to note.

Myeloid cells have important roles in inflammatory conditions. It has been shown before that BCL-2-family proteins, especially MCL-1 and A1, are induced by inflammatory stimuli like bacterial LPS. This is commonly interpreted that these stimuli and proteins operate to keep the cells alive so they can fulfil functions in host defence. Both MCL-1 and A1 have very short half-lives compared to other anti-apoptotic BCL-2-like proteins [43, 48]. A1 is strongly regulated at the transcriptional level and presumably at the translational/posttranslational level [43, 49]. As found here, A1 deficiency in responding progenitors turned LPS into a pro-apoptotic stimulus: A1 is a critical survival-promoting factor in this scenario. In A1-deficient primary BM-derived monocytes, LPS had no pro-apoptotic activity [21], similar to our model of differentiated cells. Our progenitor cells are clearly more immature. Under pro-inflammatory conditions, immature macrophage progenitor stages seem more dependent on A1.

The changes in protein expression of distinct Bcl-2 family proteins in A1-deficient cells could be interpreted as effects of altered stability due to interactions within the Bcl-2 family but could also involve compensatory regulation of other genes. Such effects have not been described for cells derived from A1-deficient mice so far. Previous analyses of protein expression were done in resting or stimulated A1-deficient B- and T-lymphocytes but did not reveal differences compared to wt and thus did not point to compensatory regulation of other pro-survival proteins or BH3-only protein Bim due to A1 deficiency [17].

NOXA is a well-established antagonist of MCL-1, and NOXA upregulation can destabilise MCL-1. Because of the similar BH3-domain binding profile of MCL-1 and A1, we tested specifically the role of NOXA in antagonising A1 in macrophages. We observed some upregulation of NOXA by LPS treatment. NOXA could be immunoprecipitated with A1; the signal was stronger when MCL-1 was antagonised, suggesting that the drug releases NOXA from MCL-1 and shifts it to A1, thereby stabilising both proteins. A1 also interacted with BIM, as detected by IP. As far as we know, this is the first demonstration of the interaction of endogenous A1 with BH3-only proteins in myeloid cells.

There was a clear reduction of A1 protein in macrophages lacking NOXA-expression; although NOXA in other cell types regulates MCL-1-stability [42], no effect of NOXA-loss on MCL-1 levels was observed. A smaller reciprocal effect was seen: NOXA levels were slightly lower in the absence of A1. NOXA therefore appears to regulate A1 levels. NOXA induces the proteasomal loss of MCL-1 through direct binding while the MCL-1 inhibitor S63845 stabilises (inactive) MCL-1. The most straightforward model is that NOXA stabilises A1 through direct binding. What this means for A1-activity is difficult to gauge and will depend on the on-off rates of the interaction. A1/Noxa interaction might indirectly prevent the neutralisation and destabilization of MCL-1. Both A1 and MCL-1 seem to be regulated by NOXA in macrophages. It will be interesting to work out the differences.

Macrophage differentiation, particularly in tissues, is exceedingly complex. The Hoxb8 system mimics the early differentiation of cells in the inflammatory monocyte/macrophage lineage [50]. The precursors are at a stage of macrophage-committed progenitors resembling common monocyte progenitors according to surface markers [35, 50, 51]. The Hoxb8 cells differentiated in GM-CSF, resemble inflammatory macrophages with some similarity to inflammatory dendritic cells [50]. Like many cell lineages, this lineage showed a very substantial change in the expression of BCL-2-family proteins. Obviously, this is tailored to suit the requirements of a given differentiation status. In macrophages, A1 clearly has its role in this network.

## Materials and methods

### Cell lines and cell culture

HoxB8 macrophage GM-SCF progenitors were derived from bone marrow of C56BL/6 wild type (wt) or genetically modified BIM^−/−^, NOXA^−/−^, BIM^−/−^NOXA^−/−^, PUMA^-/-^, BIM^-/-^PUMA^-/-^, BIM^-/-^BMF^-/-^, BAX^−/−^, BAK^−/−^, or BAX^−/−^BAK^−/−^ mice. Polyclonal macrophage progenitor cell lines were established by retroviral transduction of ER-Hoxb8 and selection in the presence of GM-CSF as described [35, 36]. Immortalised GM-CSF progenitor cells were cultured in non-cell culture-treated 6 well-plates in VLE RPMI 1640 (with stable Glutamine, with 2.0 g/L NaHCO_3_, Biochrom or PAN) supplemented with 10% FCS (Gibco, Thermo Fisher) in the presence of 5 µM oestrogen and 1% GM-CSF supernatant from GM-CSF-producing B16 cells corresponding to a final GM-SCF concentration of approximately 10 ng/ml according to Wang et al. [35] (kindly provided by Hans Häcker). Cells were kept at a maximum density of 0.5–1 × 10^6^ per well with 3 ml of medium. Cells were split every 2–3 days or the day before seeding them for an experiment or starting differentiation. Cell viability and density were assessed with the CASY cell counter (Omni Life Science). Differentiation of macrophages was induced in GM-CSF progenitors by oestrogen removal and culture in a medium containing 1% GM-SCF supernatant for 7 days (0.5 × 10^6^ progenitor cells in non-tissue culture-treated 10 cm Petri dishes in 10 ml). Differentiated cells were washed once in PBS and harvested by accutase treatment for 15 min at 37 °C.

HEK293FT cells (Invitrogen, Carlsbad, CA, USA) used for lentivirus production were cultured in tissue culture-treated plates in DMEM supplemented with 10% FCS.

The inhibitors ABT-737, ABT-199, S63845, A-1155463 (all from Selleckchem), MG-132 (Enzo Life Sciences) and Q-VD-OPH (Hycultec) were used as indicated. Since S63845 has a 6-fold lower affinity for murine than for human MCL-1 [52], concentrations of up to 10 µM were used. LPS was derived from *E. coli* O55:B5 (#tlrl-b5lps, Invivogen) and used at a concentration of 1 µg/ml. Concentrations of 100 µg/ml were also tested and gave very similar results (not shown).

### Monitoring of cell differentiation by Giemsa staining and cell surface marker expression

Staining for cellular and nuclear morphology was performed on cytospins from cultures of progenitors or differentiated macrophages or neutrophils by incubating with Giemsa solution (Merck, Darmstadt, Germany) after methanol fixation. Analysis by brightfield microscopy was performed using a Keyence BZ9000 microscope at a magnification of 20x (Keyence, Neu-Isenburg, Germany).

Expression of cell surface markers was measured after blocking unspecific binding sites with CD16/CD32 Fc-Block (BD Biosciences, San Jose, CA, USA). Cells were stained with anti-CD11b-FITC (clone M1/70, #12-0112, eBioscience, San Diego, CA, USA), anti-CD11c-PE (clone HL3, #553802, BD), Ly-6C-APC (clone HK1.4, #17-5932-82, eBioscience), anti-F4/80-AF647 (clone A3-1, #MCA497A647, Biolegend, San Diego, CA, USA), CD64-PE (clone X54-5/7.1, #139304, Biolegend), MHC-II-FITC (clone 2G9, #553623, BD), or c-Kit-APC (clone ACK2, #17-1172, eBioscience) followed by flow cytometry analysis on a FACS Fortessa (BD Biosciences, Heidelberg, Germany).

### CRISPR/Cas9-mediated genome editing

For CRISPR/Cas9-mediated genome editing, sgRNA sequences targeting all A1 isoforms were selected based on the genomic sequence of A1a using an online tool (http://crispr.mit.edu/). The following sgRNA target sequences (including PAM site) were used:

A1g7, ACAGGTACCCGCCTTTGAGT(CGG); A1g12, CTACCTTCAGTATGTGCTAC(AGG).

sgRNA sequences were cloned into the lentiCRISPRv2-puro vector (Addgene plasmid #52961) expressing both the Cas9 protein and the sgRNA [53]. CRISPR/Cas9 constructs were stably introduced into macrophage progenitors by lentiviral gene transfer. For lentivirus production, expression vectors were transfected into HEK 293FT cells, together with packaging plasmids psPAX2 and pMD2.G using Fugene HD (Roche, Mannheim, Germany). psPAX2 and pMD2.G were gifts from Didier Trono (Addgene plasmids #12260 and #12259). Lentiviral supernatants were harvested on days 2 or 3, filtered, and transduced at a cell density of 0.5–1 × 10^5^/ml in the presence of 5 μg/ml polybrene. Transduced cells were subjected to puromycin selection (5 µg/ml) after 48 h to select polyclonal knockout cell lines. Knockout efficiency was confirmed by Western blotting (Fig. S1).

### Apoptosis and cell death assays

For staining of active caspase-3, cells were washed with PBS, fixed in 2% paraformaldehyde and permeabilised with 0.5% saponin (Sigma-Aldrich). Cells were incubated with anti-active caspase-3 (Cat-Nr. # 559565, BD Pharmingen, Heidelberg, Germany) in PBS/0.5% BSA/0.5% saponin for 20 min, stained with anti-rabbit-Alexa-Fluor647 (Cat-Nr. 711-605-152, Dianova GmbH, Hamburg, Germany) for 20 min and analysed by flow cytometry.

AnnexinV-propidium iodide staining was done by washing cells with annexinV-binding buffer (eBioscience) and staining with AnnexinV-FITC (1:20; BD Pharmingen) 15 min at 4 °C. Propidium iodide (1 μg/ml; Sigma-Aldrich) was added and cells were analysed on a FACS Calibur (Becton Dickinson, Heidelberg, Germany). Viability was determined as the percentage of AnnexinV/PI-negative cells (cells negative for both AnnexinV and PI) of all cells analysed. Samples were analysed by flow cytometry on a FACS Calibur. In some experiments, cell death was measured as the loss of cell membrane integrity by staining with live-dead fixable Far Red (Thermo Fisher). Cells were stained for 30 min on ice, washed with PBS, fixed with 2% paraformaldehyde and analysed by flow cytometry.

Data analysis was performed with FlowJo 10 software.

### Phagocytosis assay

Day 8 differentiated CTRL or A1-deficient macrophages were co-incubated in suspension with pHrodo Red *E. coli* bioparticles (100 µg/ml; Thermo Fisher Scientific) for 90 min at 37 °C. Cells were washed once with ice-cold PBS and analysed for phagocytosed *E. coli* particles by flow cytometry. As specificity controls, samples were treated the same way with *E. coli* bioparticles but were left on ice during the whole incubation time. Data are representative of two independent experiments.

### Analysis of reactive oxygen species (ROS) production

Day 8 differentiated CTRL or A1-deficient macrophages were stimulated with PMA (5 µg/ml; Sigma-Aldrich) for 60 min. Their capacity to generate (ROS) was assessed by the addition of the ROS indicator Dihydrodamine 123 (DHR123, 2.5 µM; Hycultec) for the last 30 min of incubation. Cells were placed on ice, washed once with ice-cold PBS and analysed by flow cytometry.

### Immunoblot analysis

Cells were harvested with accutase treatment (20 min 37 °C, differentiated macrophages), or harvested by repeated pipetting (progenitors). After washing once with PBS, cells were lysed directly in 1x Blot LDS Sample buffer and boiled at 70 °C for 10 min (Bolt gels and Bis-Tris gels) or in Laemmli buffer and boiled at 95 °C (TGX gels). Extracts were separated by SDS-PAGE on 4–12% Bolt Bis-Tris Precast gels (Invitrogen), 4–12% Criterion XT Bis-Tris Gels (Bio-Rad) or 4–20% or 8–16% Criterion TGX Precast gels (Bio-Rad). Proteins were transferred onto PVDF or Nitrocellulose membranes (0.2 μm) by wet transfer overnight or by Turboblot transfer (Bio-Rad). Membranes were probed with antibodies against murine A1 (clone 6D6, gift from Marco Herold, WEHI, Melbourne, Australia), MCL-1 (#600-401-394, Rockland), BCL-2 (clone 3F11, BD), BCL-X_L_ (clone 54H6, Cell Signaling), BCL-w (clone 31H4, Cell Signaling) BAX (#2772, Cell Signaling), BAK (#06-536, Milipore), BIM (clone C34C5, Cell Signaling) NOXA (#ab36833, Abcam), PUMA (clone E1S7A, Cell Signaling) and GAPDH (#MAB384, Merck/Millipore). Proteins were visualised using peroxidase-conjugated anti-rabbit IgG (#A6667, Sigma), anti-rabbit-IgG-Fc (#AP156P, Millipore Upstate), anti-mouse IgG (#205-035-108, Dianova), anti-hamster IgG (#127-035-160, Dianova) or anti-rat IgG (#112-035-062, Dianova) antibodies by enhanced chemoluminescence detection (ECL Prime, Cytiva, Freiburg, Germany; SuperSignal West Femto or Pico Substrate, Pierce, Thermo Fisher Scientific, Schwerte, Germany). In some cases, blots were stripped before reprobing using the Restore™ PLUS Western Blot Stripping Buffer (Thermo Fisher Scientific) or a stripping buffer containing 20 mM Tris-HCl pH 7.5, 6 M Gn-HCl, 0.2% Nonidet-P-40 (NP-40), 0.1 M beta-mercaptoethanol.

### Quantitative real-time RT-PCR

For this, 1 × 10^6^ cells were harvested, washed once with PBS and lysed in 300 µl Tri Reagent (Zymed Research). RNA was isolated using the Direct-zol RNA Miniprep Kit (Zymed Research) according to the manufacturer’s instructions. Then, 500–1000 ng of total RNA were reverse-transcribed into cDNA using the RevertAid cDNA synthesis kit (Thermo Fisher, K1622) with random hexamer primers according to the manufacturer’s instructions. qPCR was performed using the SYBR Select Master Mix (Thermo Fisher) on a Quant Studio 5 Real-Time PCR System (Thermo Fisher). Relative mRNA-expression levels were normalised to actin as a reference gene. Fold change was calculated after normalisation to the uninfected CTRL sample. Primers originally published by Sochalska et al. [54] were as follows: *A1* fwd: 5´-CCT GGC TGA GCA CTA CCT TC-3´ rev: 5´-TCC ACG TGA AAG TCA TCC AA-3´; *Mcl1* fwd: 5´-TAA CAA ACT GGG GCA GGA TT-3´, rev: 5´-GTC CCG TTT CGT CCT TAC AA-3´; *Noxa* fwd: 5´-CCC ACT CCT GGG AAA GTA-3´, rev: 5´-AAT CCC TTC AGC CCT TGA TT-3´; *Actin* fwd: 5´- ACT GGG ACG ACA TGG AGA AG, rev: 5´-GGG GTG TTG AAG GTC TCA AA-3´.

### Immunoprecipitation

Cell pellets (15 × 10^6^ cells per sample for progenitors or 5.5 × 10^6^ cells per sample for differentiated macrophages) were lysed in 300 µl 1% CHAPS lysis buffer (20 mM Tris pH 7.4, 135 mM NaCl, 1.5 mM MgCl_2_, 1 mM EGTA, 1% CHAPS, 10% glycerol, 2x protease inhibitors (Roche), 1 mM PMSF (Sigma-Aldrich)) for 15 min on ice. Samples were centrifuged for 15 min (20,900×*g*), and supernatants were used for IP. A1 antibody (rat monoclonal, gift from Dr. Marco Herold, WEHI, Melbourne) [55] or MCL-1 antibody (rabbit monoclonal, clone Y37, Epitomics, Abcam #ab32087) was added to the lysates together with 30 µl of agarose G beads (50% slurry, Millipore, #11719416001) and samples were incubated in Mobicol classic columns (3 µm filters) (MoBiTec) for 4 h at 4 °C with overhead rotation. Samples were then centrifuged (2000×*g*), unbound fractions of the A1 IP were used for further Mcl-1 IPs and agarose bead pellet was washed once with 20 ml 1% CHAPS buffer, then 3 times with 400 µl 1% CHAPS buffer (including protease inhibitors); (co-)precipitated proteins were eluted with 50 µl 2x Laemmli buffer at 95 °C for 10 min. Input samples were loaded as 1/45 of the eluate fraction. Proteins were separated by SDS-PAGE on 8–16% Criterion TGX Precast Midi Gels (Bio-Rad), transferred onto 0.2 µm PVDF membranes and detected as described above.

### Statistical analysis

Statistical analysis was performed with Prism (V8, GraphPad) using two-way ANOVA and Sidak’s, Tukey’s or Dunnett’s multiple comparison tests for multiple testing. Bars represent the mean, and error bars show the standard error of the mean. At least three independent biological replicates were chosen as sample size for each analysis.

## Supplementary information

Supplementary figures and figure legends S1-S7

Original Western Blots uncropped

## Data Availability

The datasets used and/or analysed during the current study are available from the corresponding author upon reasonable request.
