# Supplementary material for: Contribution of A1 to macrophage survival in cooperation with MCL-1 and BCL-XL in a murine cell model of myeloid differentiation
Source: Cell Death Dis. 2024 Sep 16;15(9):677. doi: 10.1038/s41419-024-07064-z (PMC11405755; doi:10.1038/s41419-024-07064-z)
Supplement: Supplementary file 1 — Supplementary figures and figure legends S1-S7 [file 41419_2024_7064_MOESM1_ESM.pdf]

Fig. S1

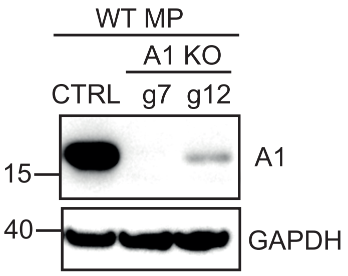

Fig. S2

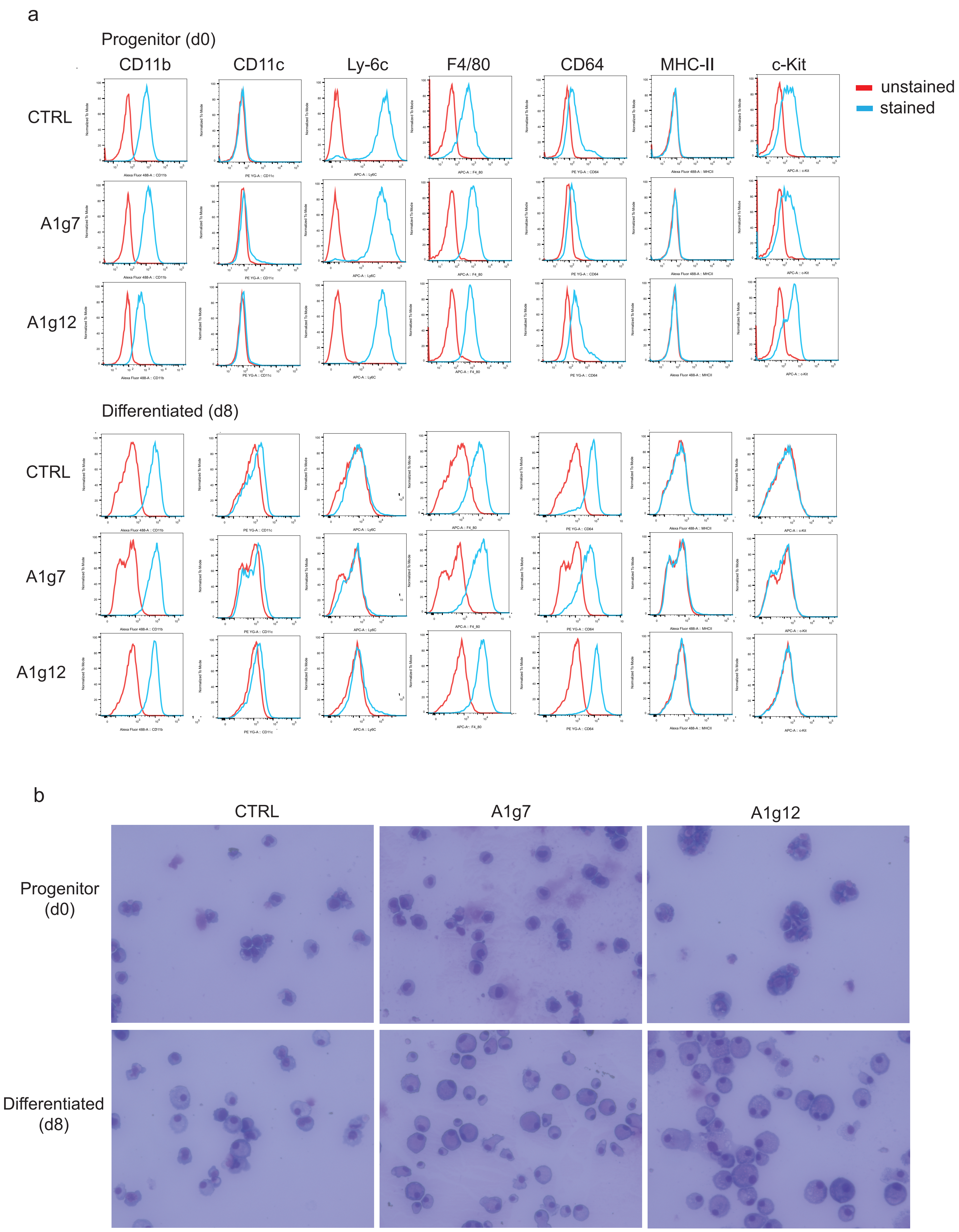
a
Progenitor (d0)
CD11b
CD11c
Ly-6c
F4/80
CD64
MHC-II
c-Kit
unstained
stained
CTRL
A1g7
A1g12
Differentiated (d8)
CTRL
A1g7
A1g12
b
CTRL
A1g7
A1g12
Progenitor (d0)
Differentiated (d8)

Fig. S2

c

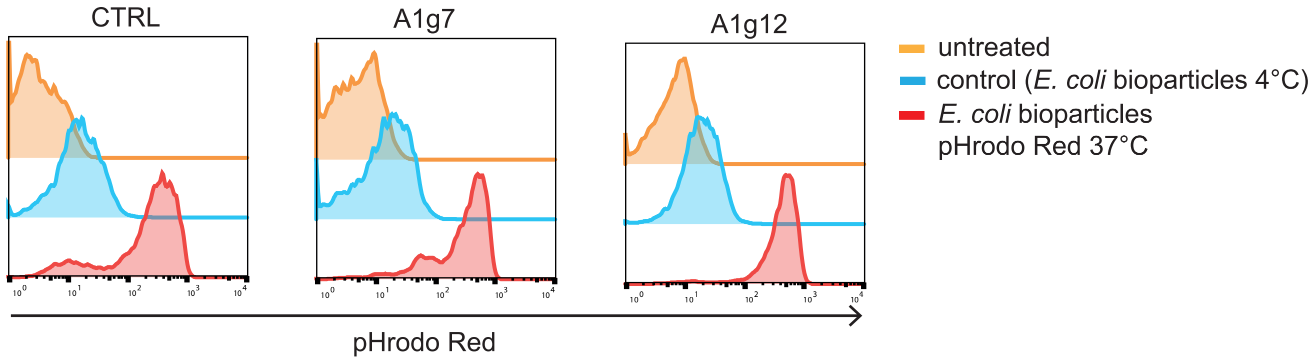

CTRL
A1g7
A1g12
untreated
control (*E. coli* bioparticles 4°C)
*E. coli* bioparticles
pHrodo Red 37°C
pHrodo Red


d

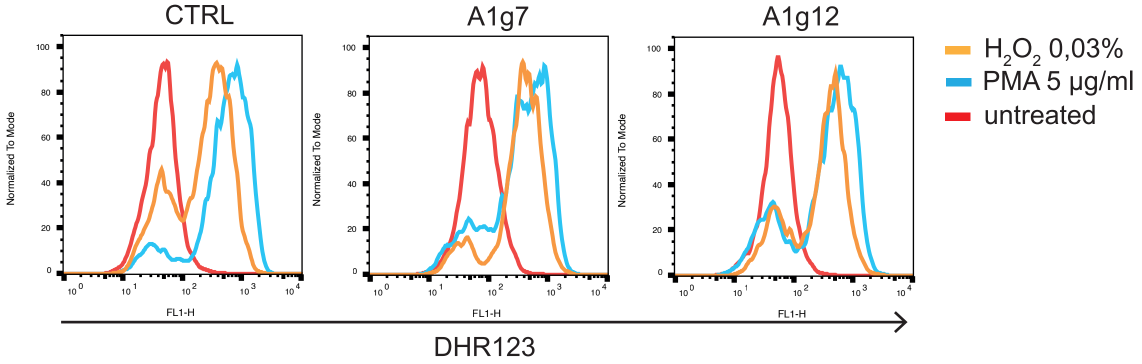

CTRL
A1g7
A1g12
Normalized To Mode
FL1-H
$H_2O_2$ 0,03%
PMA 5 µg/ml
untreated
DHR123

Fig. S3

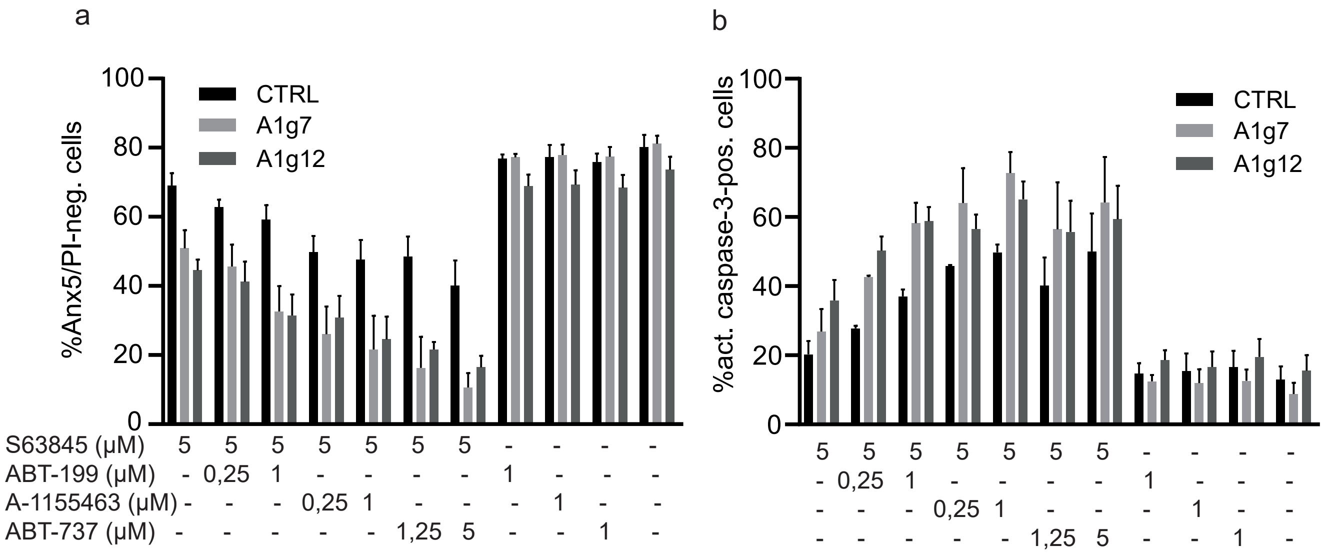
a
%Anx5/PI-neg. cells
100
80
60
40
20
0
CTRL
A1g7
A1g12
S63845 (µM) 5 5 5 5 5 5 5 - - - -
ABT-199 (µM) - 0,25 1 - - - - 1 - - -
A-1155463 (µM) - - - 0,25 1 - - - 1 - -
ABT-737 (µM) - - - - - 1,25 5 - - 1 -
b
%act. caspase-3-pos. cells
100
80
60
40
20
0
CTRL
A1g7
A1g12
5 5 5 5 5 5 5 - - - -
- 0,25 1 - - - - 1 - - -
- - - 0,25 1 - - - 1 - -
- - - - - 1,25 5 - - 1 -

Fig. S4

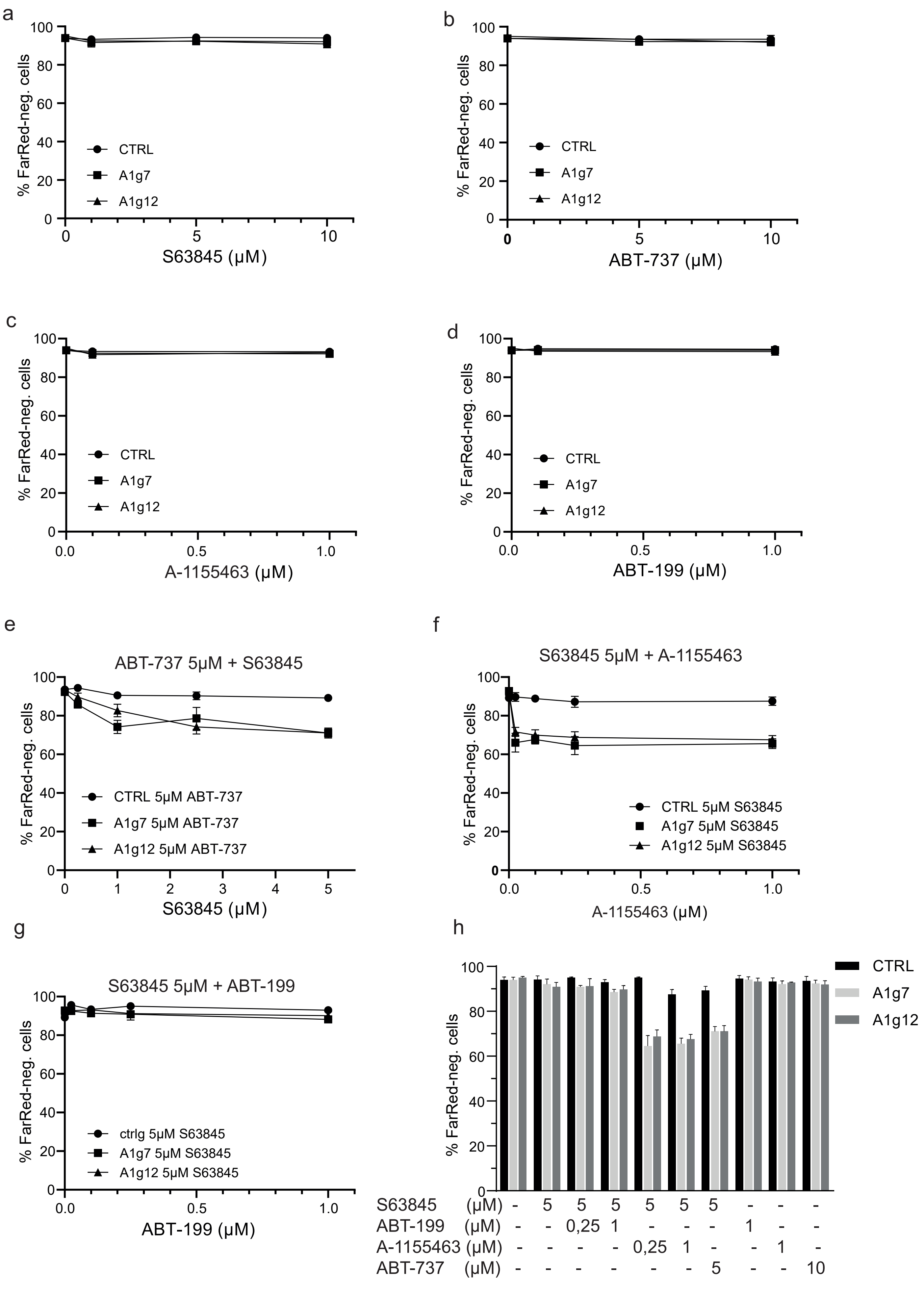
a
% FarRed-neg. cells
CTRL
A1g7
A1g12
S63845 (µM)
b
% FarRed-neg. cells
CTRL
A1g7
A1g12
ABT-737 (µM)
c
% FarRed-neg. cells
CTRL
A1g7
A1g12
A-1155463 (µM)
d
% FarRed-neg. cells
CTRL
A1g7
A1g12
ABT-199 (µM)
e
ABT-737 5µM + S63845
% FarRed-neg. cells
CTRL 5µM ABT-737
A1g7 5µM ABT-737
A1g12 5µM ABT-737
S63845 (µM)
f
S63845 5µM + A-1155463
% FarRed-neg. cells
CTRL 5µM S63845
A1g7 5µM S63845
A1g12 5µM S63845
A-1155463 (µM)
g
S63845 5µM + ABT-199
% FarRed-neg. cells
ctrlg 5µM S63845
A1g7 5µM S63845
A1g12 5µM S63845
ABT-199 (µM)
h
% FarRed-neg. cells
CTRL
A1g7
A1g12
S63845 (µM) - 5 5 5 5 5 5 - - -
ABT-199 (µM) - - 0,25 1 - - - 1 - -
A-1155463 (µM) - - - - 0,25 1 - - 1 -
ABT-737 (µM) - - - - - - 5 - - 10

Fig. S4

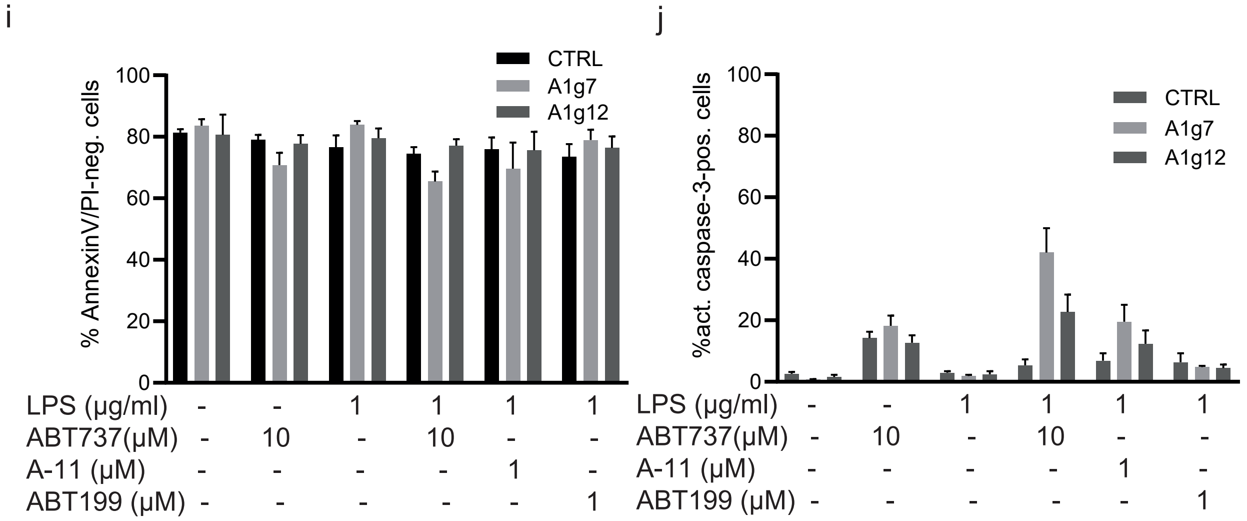
i
% AnnexinV/PI-neg. cells
100
80
60
40
20
0
CTRL
A1g7
A1g12
LPS (µg/ml) - - 1 1 1 1
ABT737(µM) - 10 - 10 - -
A-11 (µM) - - - - 1 -
ABT199 (µM) - - - - - 1
j
%act. caspase-3-pos. cells
100
80
60
40
20
0
CTRL
A1g7
A1g12
LPS (µg/ml) - - 1 1 1 1
ABT737(µM) - 10 - 10 - -
A-11 (µM) - - - - 1 -
ABT199 (µM) - - - - - 1

Fig. S5

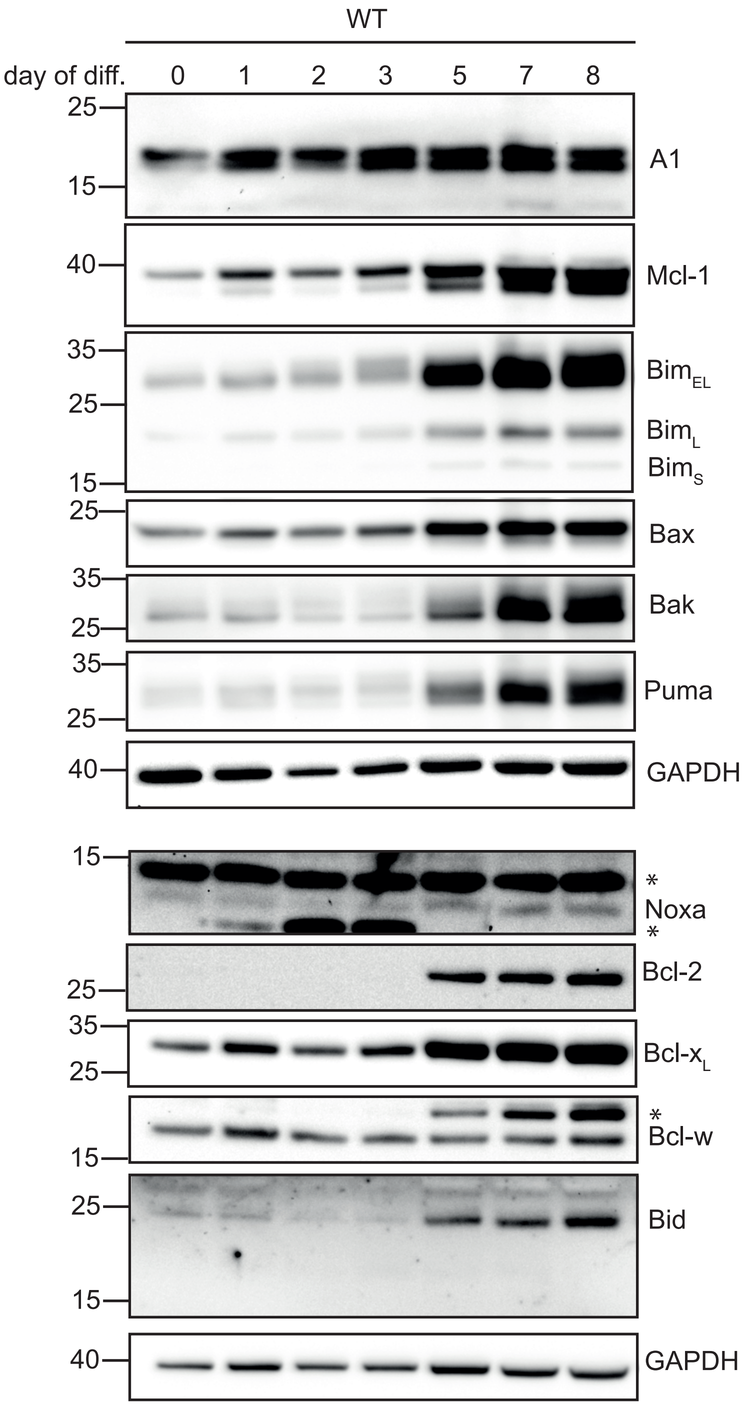
WT
day of diff.
0
1
2
3
5
7
8
25
15
A1
40
Mcl-1
35
25
15
Bim$_{EL}$
Bim$_{L}$
Bim$_{S}$
25
Bax
35
25
Bak
35
25
Puma
40
GAPDH
15
*
Noxa
*
Bcl-2
25
35
25
Bcl-x$_{L}$
*
Bcl-w
15
25
15
Bid
40
GAPDH

Fig. S6

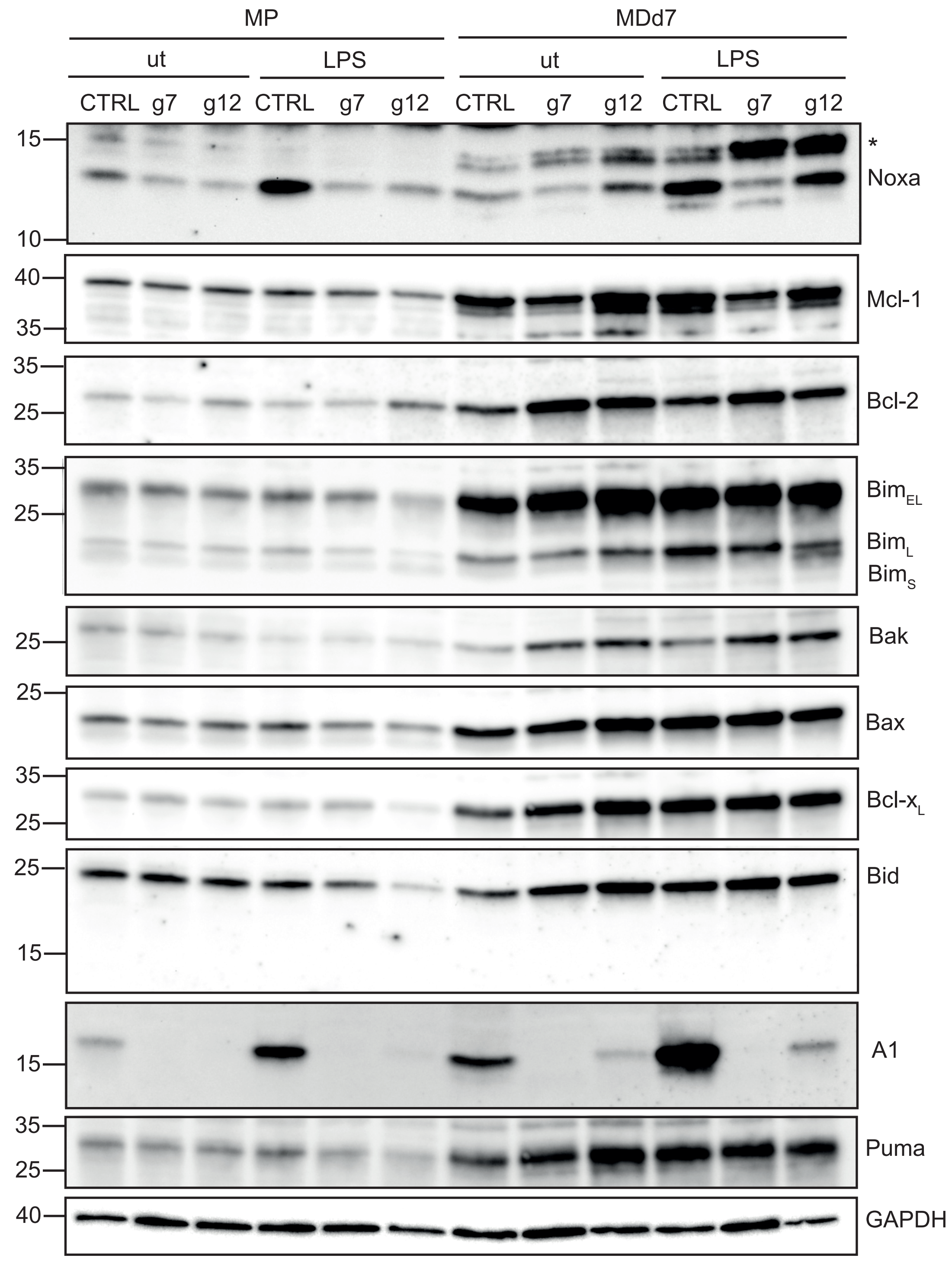
MP
MDd7
ut
LPS
ut
LPS
CTRL g7 g12 CTRL g7 g12 CTRL g7 g12 CTRL g7 g12
15
10
*
Noxa
40
35
Mcl-1
35
25
Bcl-2
35
25
BimEL
BimL
BimS
25
Bak
25
Bax
35
25
Bcl-xL
25
15
Bid
15
A1
35
25
Puma
40
GAPDH

Fig. S7

a

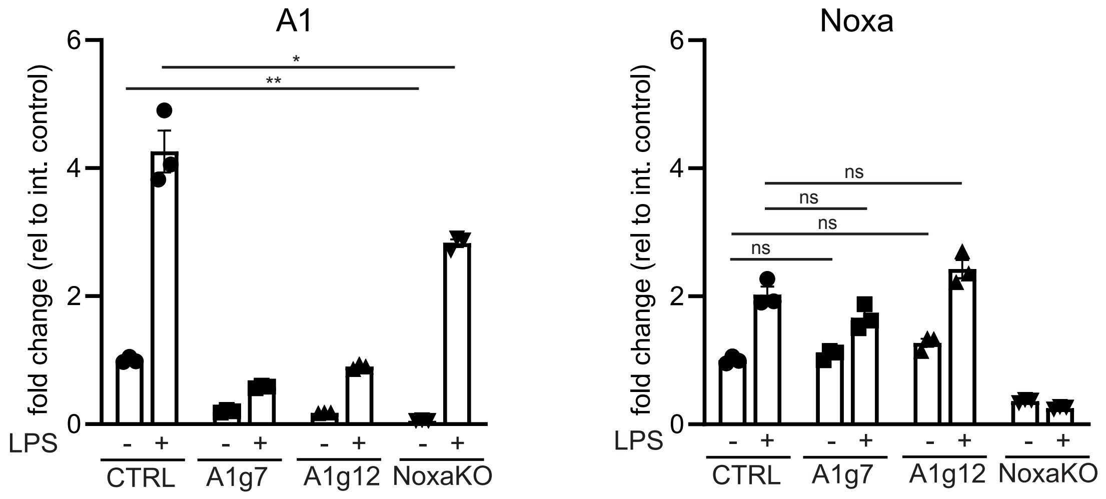
A1
Noxa
fold change (rel to int. control)
0
2
4
6
*
**
ns
ns
ns
ns
LPS
-
+
CTRL
A1g7
A1g12
NoxaKO

b

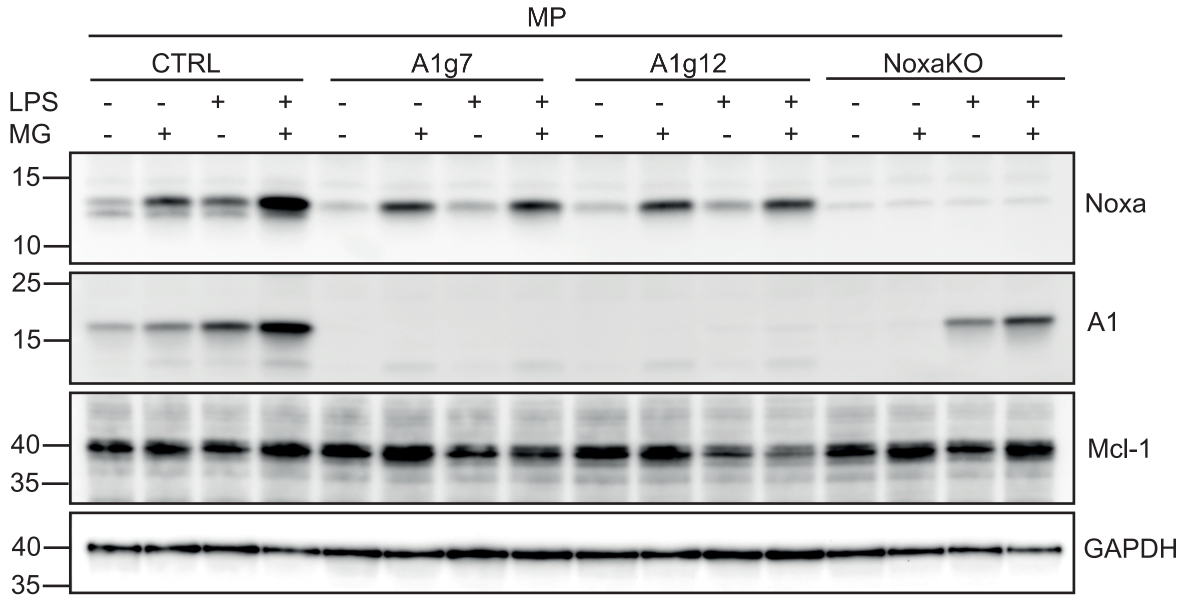
MP
CTRL
A1g7
A1g12
NoxaKO
LPS
MG
15
10
25
15
40
35
40
35
Noxa
A1
Mcl-1
GAPDH

## Supplementary figure legends

**Fig. S1: Confirmation of A1-deletion in macrophage progenitors by Western blot.**

Macrophage progenitors were directly lysed in Bolt sample buffer. Samples were subjected to SDS-PAGE and transferred to nitrocellulose. Membrane was probed with anti-mouse A1 (gift from Marco Herold). GAPDH served as loading control.

**Fig. S2: Surface marker expression, morphology and functional analysis of A1-deficient progenitors and differentiated macrophages is comparable to wt CTRL cells**

a, WT CTRL and A1-deficient macrophage progenitors and day 7 differentiated macrophages were stained with fluorochrome-conjugated antibodies against various surface markers (CD11b, CD11c, Ly-6c, F4/80, CD64, MHC-II, c-Kit) and analysed by flow cytometry (FACS Fortessa). Data are representative of 2-3 independent experiments.

b, WT CTRL and A1-deficient progenitors and day 8 differentiated macrophages were collected by cytospin on glass slides and stained with Giemsa to monitor changes of cell morphology upon differentiation. Samples were analysed by bright field microscopy (Keyence BZ-9000) at a magnification of 40x. Data are representative of 2 independent experiments.

c, Analysis of phagocytic capacity. Day 8 differentiated CTRL or A1-deficient macrophages were co-incubated with pHrodo Red *E. coli* bioparticles (100µg/ml) for 90 min at 37°C. Cells were washed once with ice-cold PBS and analysed for phagocytosed *E. coli* particles by flow cytometry. Specificity controls included untreated samples and samples containing E. coli bioparticles left on ice during the incubation time. Data are representative of two independent experiments.

d, Analysis of reactive oxygen species. Day 8 differentiated CTRL or A1-deficient macrophages were stimulated with PMA (5 µg/ml) for 60min. Their capacity to generate reactive oxygen species (ROS) was assessed by addition of the ROS indicator Dihydrodamine 123 (DHR123, 2,5 µM) for the last 30 min of incubation. Cells were placed on

ice, washed once with ice-cold PBS and analysed by flow cytometry. Data are representative of two independent experiments.

**Fig. S3: Targeting anti-apoptotic proteins in macrophage progenitors reveals an essential role of MCL-1 in cooperation with A1 and BCL-$X_L$**

a,b Hoxb8 macrophage progenitors were treated for 4 hours with the following single or combined inhibitors targeting anti-apoptotic BCL-2-family proteins, ABT-737 (specific for BCL-$X_L$ and BCL-2), S63845 (specific for MCL-1), ABT-199 (specific for BCL-2) or A-1155463 (specific for BCL-$X_L$) at the concentrations indicated. Cell death was assessed by AnnexinV/PI staining (a) or active Caspase-3 staining (b) and flow cytometry. Data are means/SEM of 3 independent experiments.

**Fig. S4: Differentiation confers resistance to inhibition of single anti-apoptotic proteins which can be overcome by combined neutralisation of A1, MCL-1 and BCL-$X_L$.**

A1-deficient or CTRL GM-CSF Hoxb8 cells (A1g7, A1g12 or CTRL) were differentiated into macrophages for 7 days by oestrogen withdrawal.

a-d, Differentiated macrophages were treated for 4 hours with the following inhibitors targeting anti-apoptotic BCL-2-family proteins: ABT-737 (a, specific for BCL-$X_L$ and BCL-2), S63845 (b, specific for MCL-1), ABT-199 (c, specific for BCL-2) or A-1155463 (d, specific for BCL-$X_L$) at the concentrations (µM) indicated. e-h, Differentiated macrophages were treated as above with single or combined inhibitors as indicated. DMSO served as solvent control. Cell death as measured by loss of cell membrane integrity was assessed by Live-Dead staining using Live-Dead-FarRed (Thermo Fisher) and flow cytometry (FACS Calibur). Data are means/SEM of 3-7 independent experiments.

i, j, Differentiated macrophages were were co-treated with LPS and single or combined inhibitors against BCL-$X_L$/BCL-2 (ABT-737), MCL-1 (S63845), BCL-2 (ABT-199) or BCL-$X_L$ (A-1155463) at the concentrations indicated for 4 hours. DMSO served as solvent control. Viability and apoptotic cell death were assessed in parallel by AnnexinV/PI staining (i) and

staining against active caspase-3 (j) followed by flow cytometry (FACS Calibur). Data are means/SEM of 3 independent experiments.

**Fig. S5: Expression of BCL-2-family members during differentiation of Hoxb8 macrophage progenitors induced by oestrogen withdrawal**

WT CTRL macrophage progenitors were induced to undergo differentiation by oestrogen withdrawal for up to 8 days. Cells were harvested at the timepoints indicated, directly lysed in Bolt sample buffer, subjected to SDS-PAGE and proteins were transferred onto nitrocellulose membranes. Blots were probed for antibodies against various BCL-2-family members as indicated. GAPDH served as loading control. Asterisk denotes unspecific bands. Data are representative of at least 2 independent experiments.

**Fig. S6: Expression of BCL-2-family members in progenitors and differentiated macrophages upon LPS stimulation**

WT CTRL or A1-deficient progenitors or differentiated macrophages were stimulated with LPS (1 µg/ml) or left untreated. Cell were harvested after 4 hours, directly lysed in Bolt sample buffer, subjected to SDS-PAGE and proteins were transferred onto nitrocellulose mebranes. Blots were probed for antibodies against various BCL-2-family members as indicated. GADPH served as loading control. Asterisk denotes unspecific bands. Data are representative of 2 independent experiments.

**Fig. S7: mRNA-expression levels and protein stability of A1 and NOXA**

a, WT CTRL, A1- or NOXA-deficient progenitors were stimulated with LPS (1 µg/ml) or left untreated for 4 hours and subjected to quantitative RT-PCR. Shown are relative mRNA-expression levels of A1 (left) and NOXA (right) normalized to actin as a reference gene. Fold change was calculated after normalization to the uninfected CTRL sample. Data are means/SEM of 3 independent experiments. qPCRs were performed in technical duplicates

for each sample. Statistical analysis was performed using two-way ANOVA, corrected for multiple comparisons by the Sidak method.

b, WT CTRL or A1-deficient progenitors were stimulated with LPS (1 µg/ml) or left untreated for 4 hours. In some samples, MG-132 (10 µM) was added for the last hour of stimulation. Cells were directly lysed in Bolt sample buffer, subjected to SDS-PAGE and proteins were transferred onto PVDF menbranes. Blots were probed for antibodies against A1, MCL-1 and NOXA as indicated. GADPH served as loading control. Data are representative of 2 independent experiments.
