# Supplementary material for: Contribution of A1 to macrophage survival in cooperation with MCL-1 and BCL-XL in a murine cell model of myeloid differentiation
Source: Cell Death Dis. 2024 Sep 16;15(9):677. doi: 10.1038/s41419-024-07064-z (PMC11405755; doi:10.1038/s41419-024-07064-z)

Original Western blots uncropped

related to Fig.7

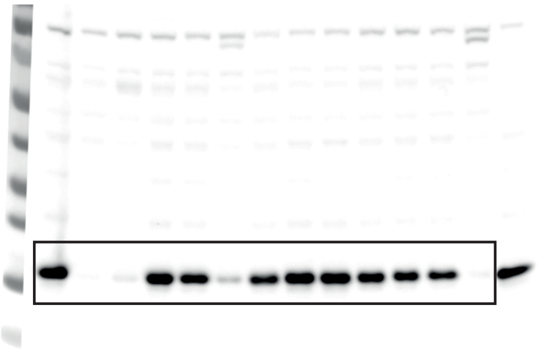

A1

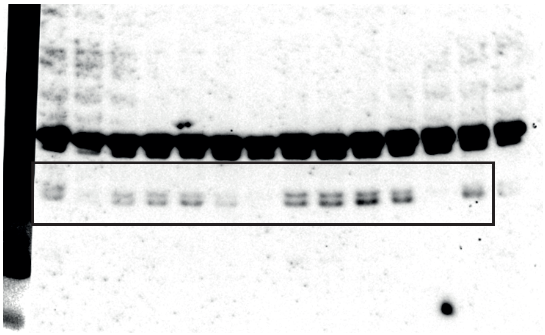

Puma

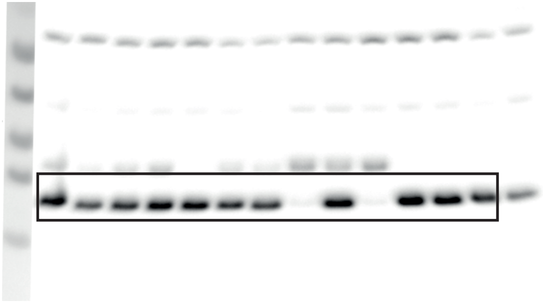

Bax

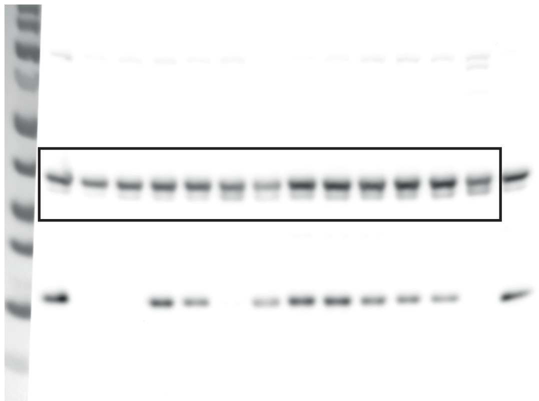

Mcl-1

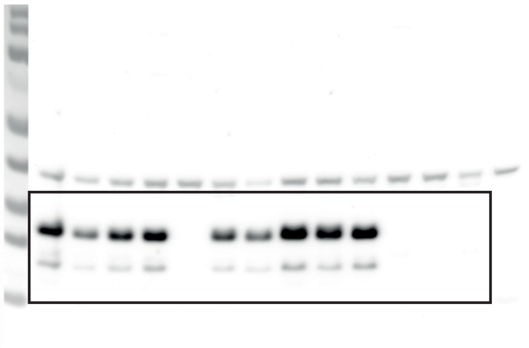

Bim_{EL}

Bim_{L}

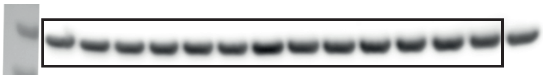

GAPDH

Original Western blots uncropped

related to Fig.7

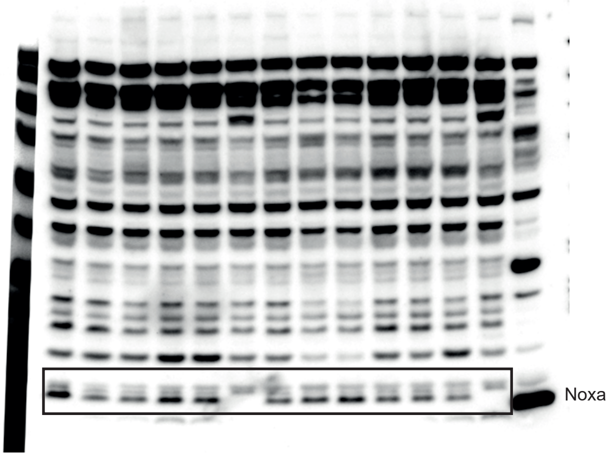

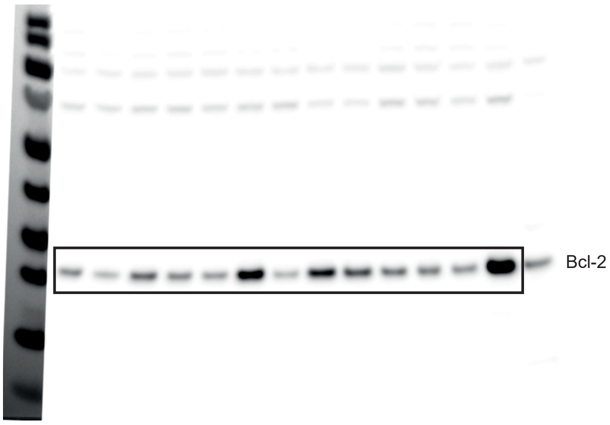

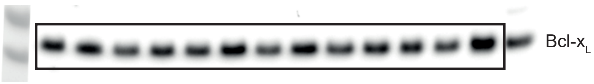

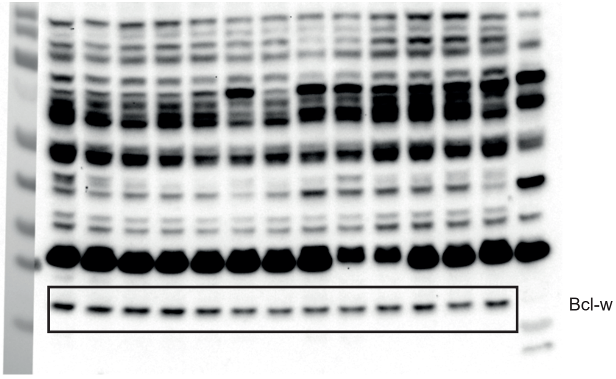

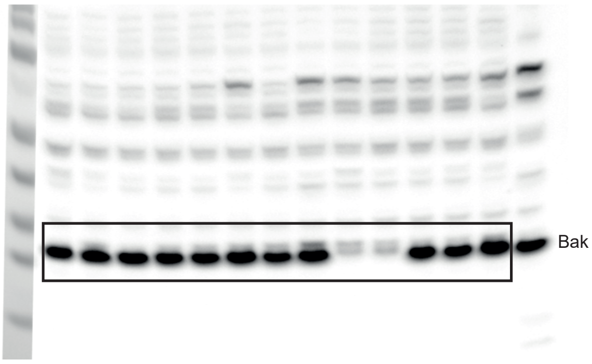

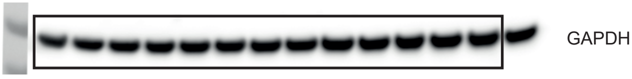

Original Western blots uncropped

related to Fig.8a

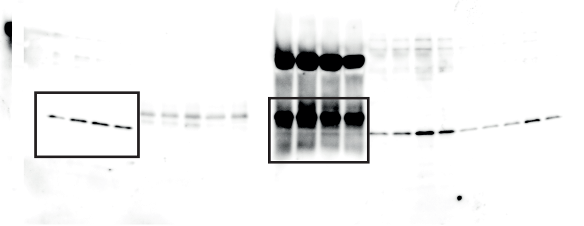

Bak

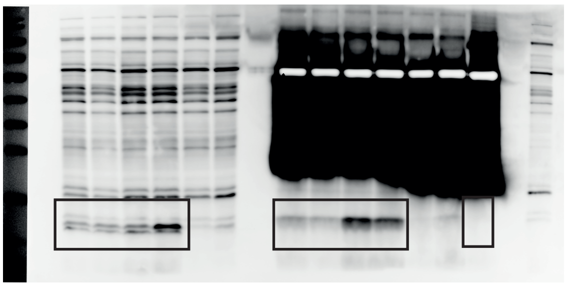

Noxa

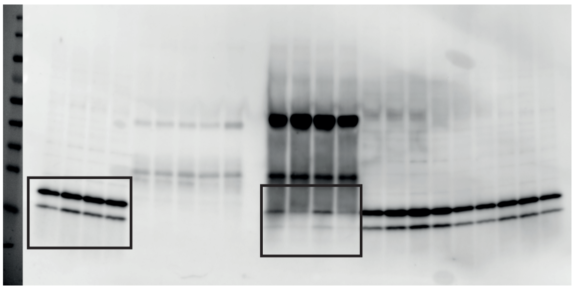

Bax

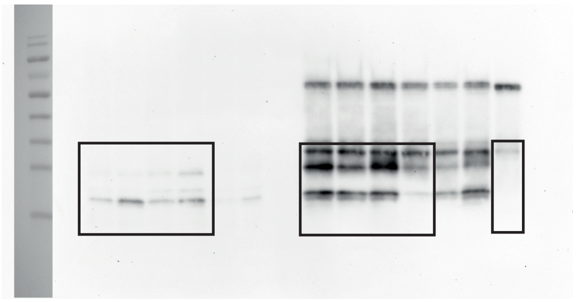

Bim

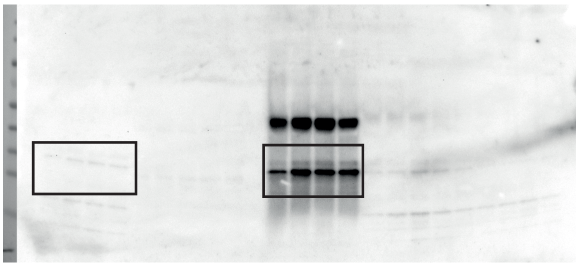

Mcl-1

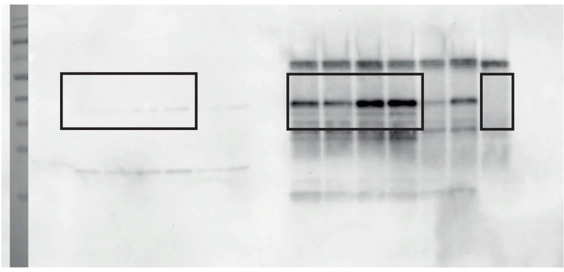

Mcl-1

Original Western blots uncropped

related to Fig. 8b

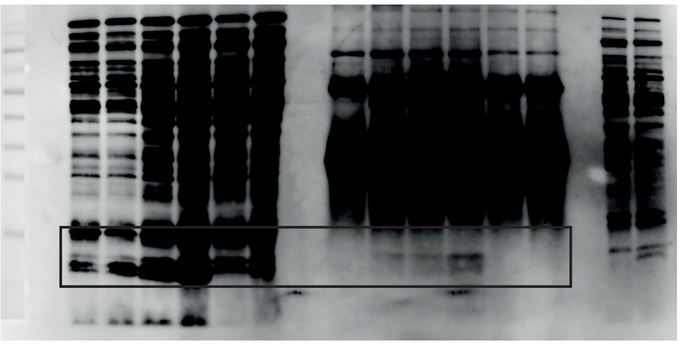

Noxa

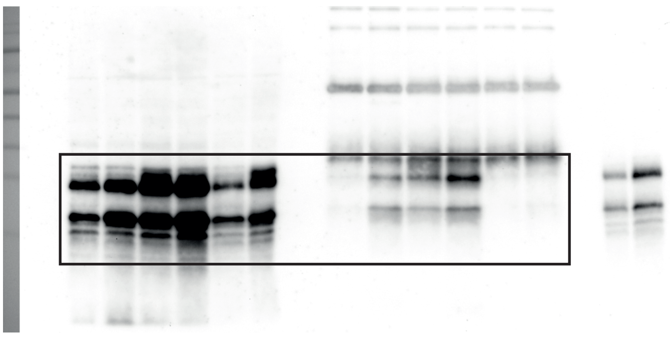

Bim

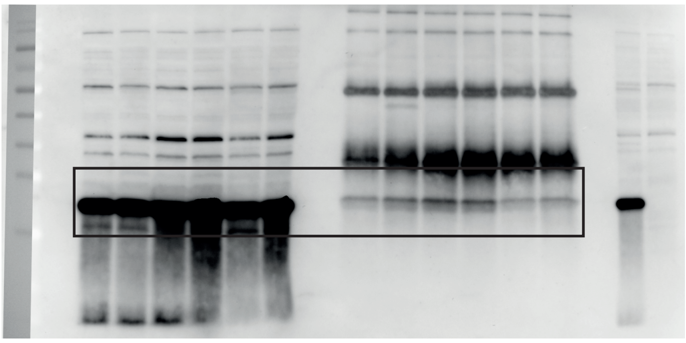

Bax

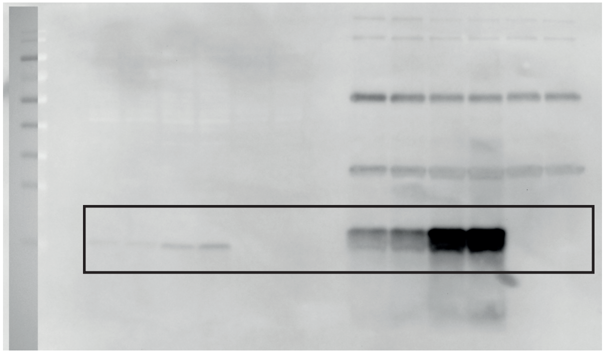

A1

Original Western blots uncropped

related to Fig.8c

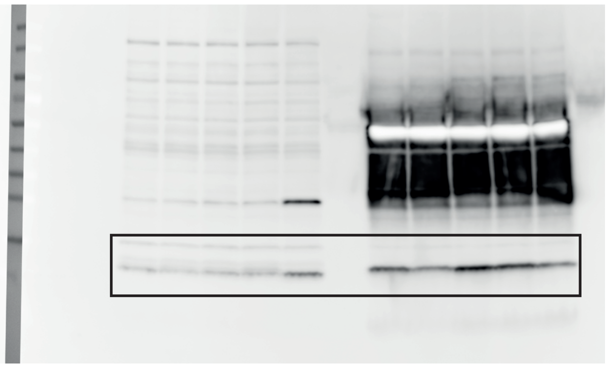

Noxa

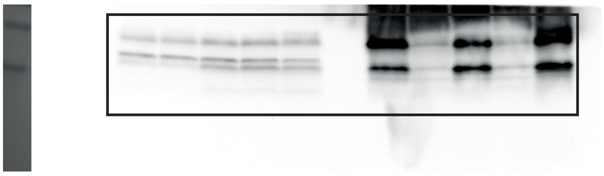

Bim_{EL}
Bim_{L}

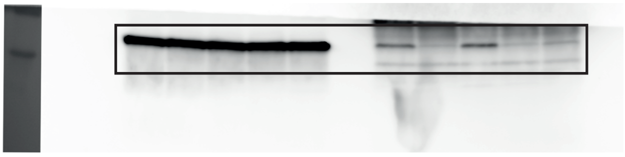

Bax

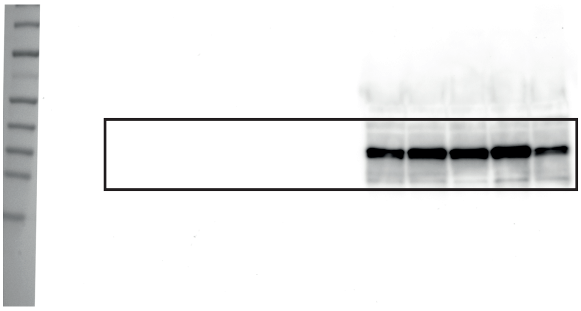

Mcl-1

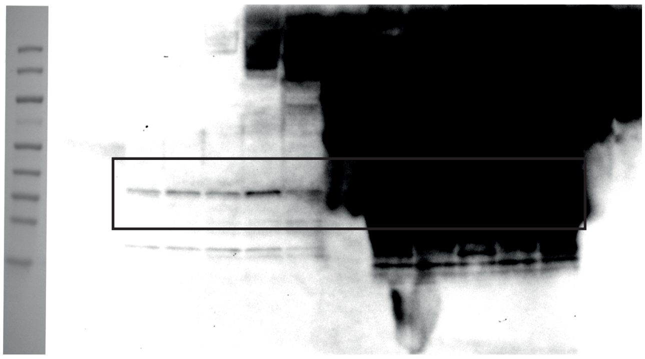

Mcl-1

Original Western blots uncropped

related to Fig.8d

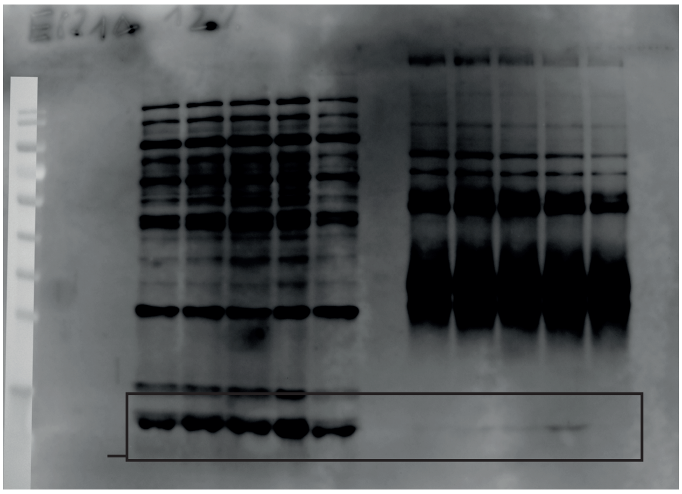

Noxa

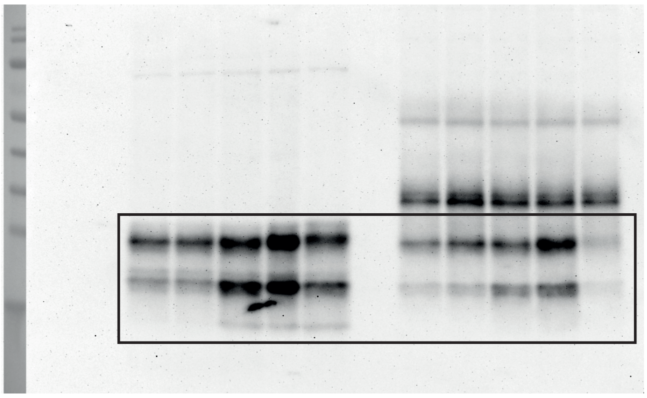

Bim_{EL}

Bim_{L}

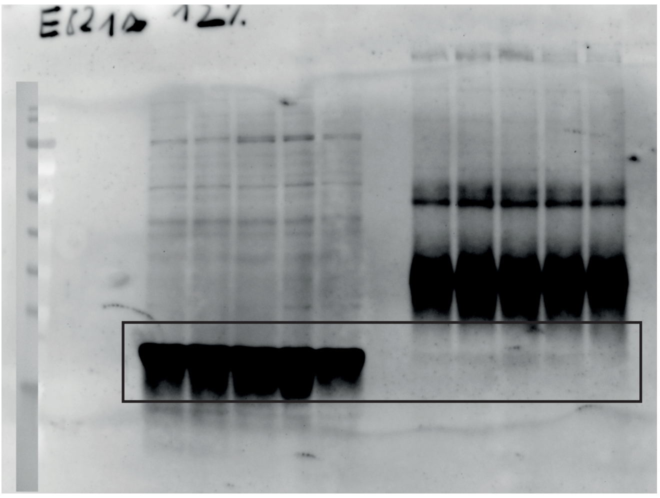

Bax

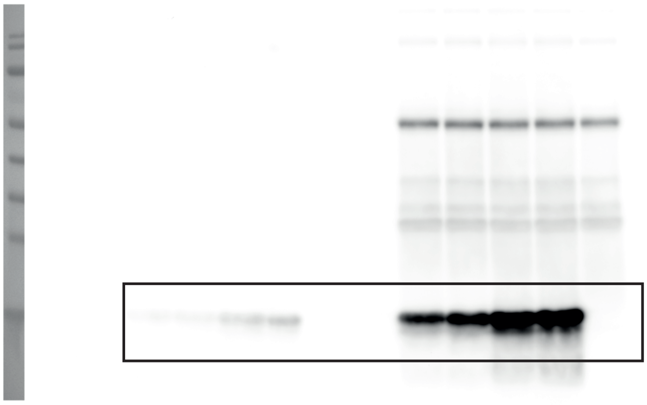

A1

Original Western blots uncropped

related to Fig. S1

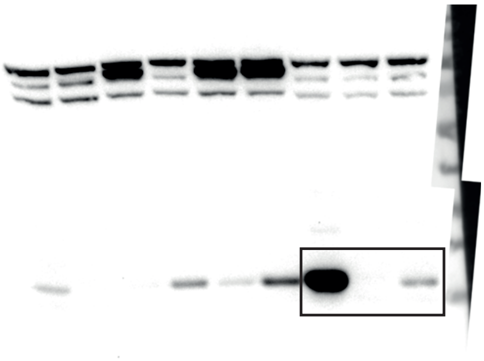

A1

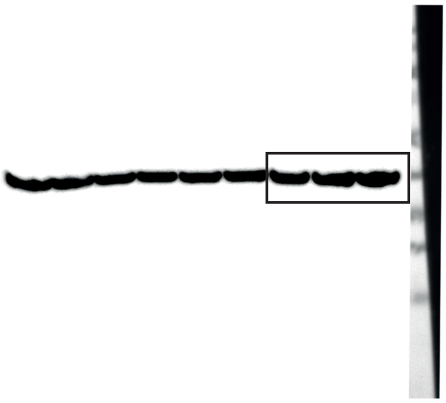

GAPDH

Original Western blots uncropped

related to Fig. S3

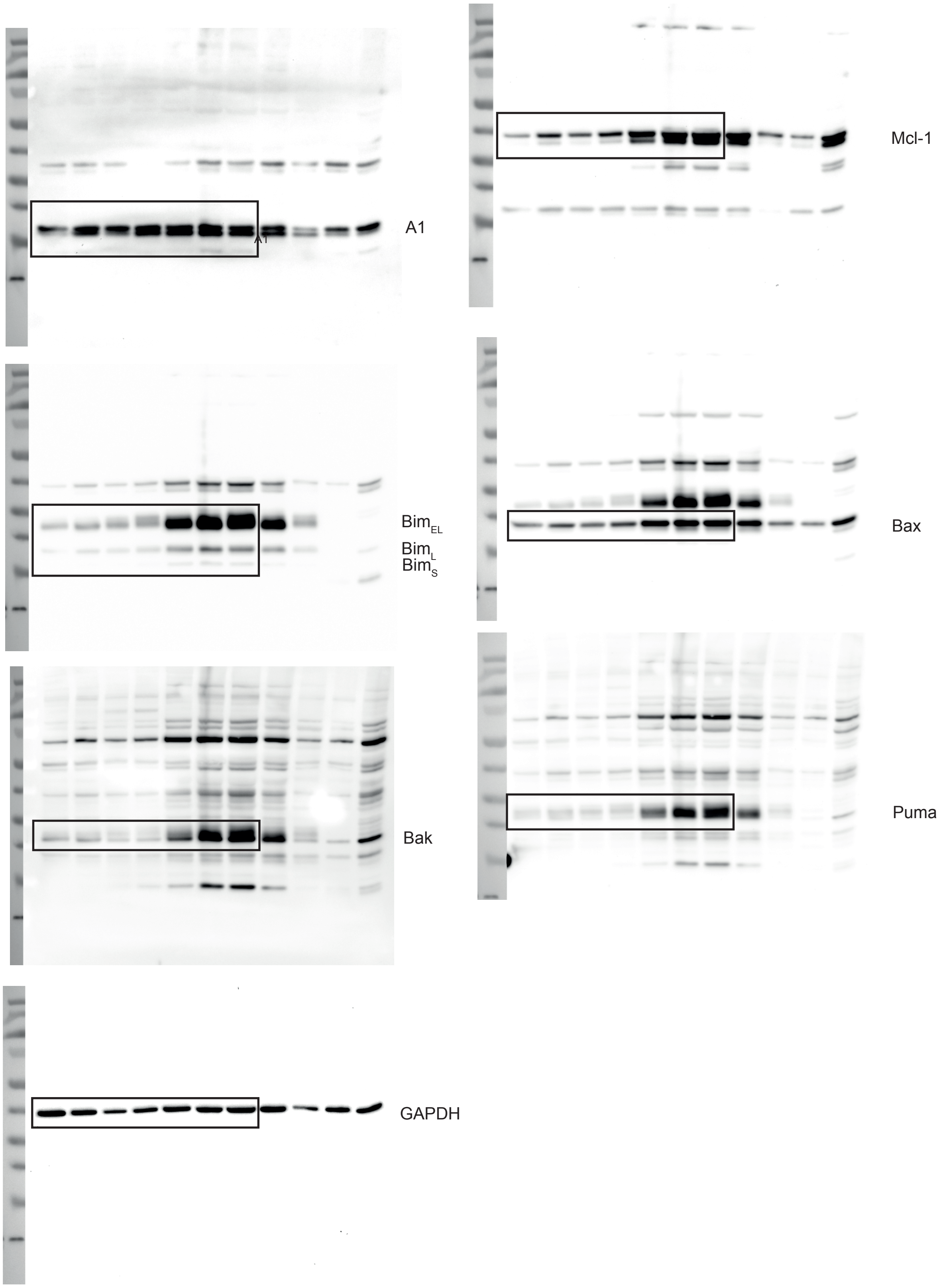

Original Western blots uncropped

related to Fig. S3

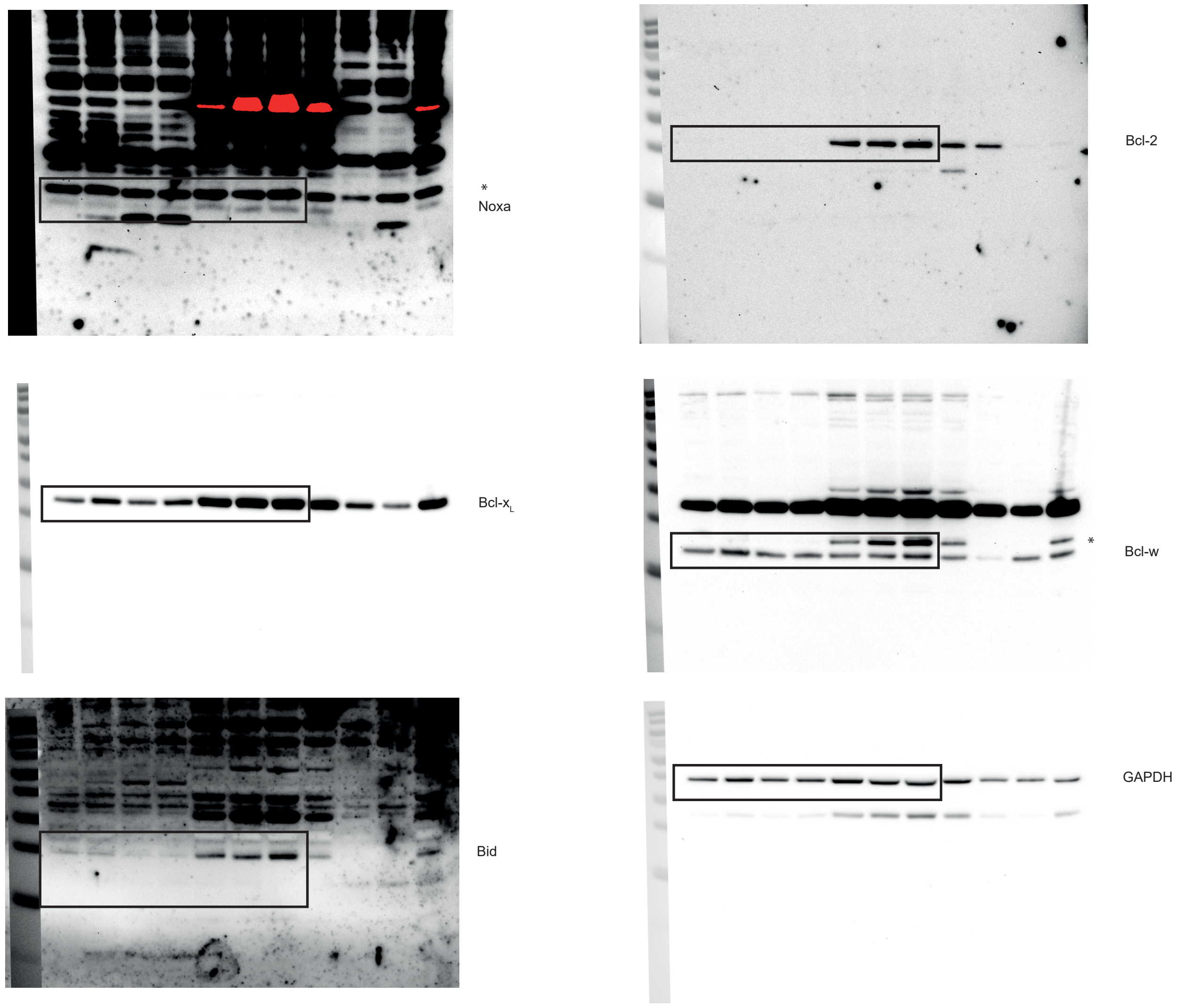

Original Western blots uncropped

related to Fig. S4

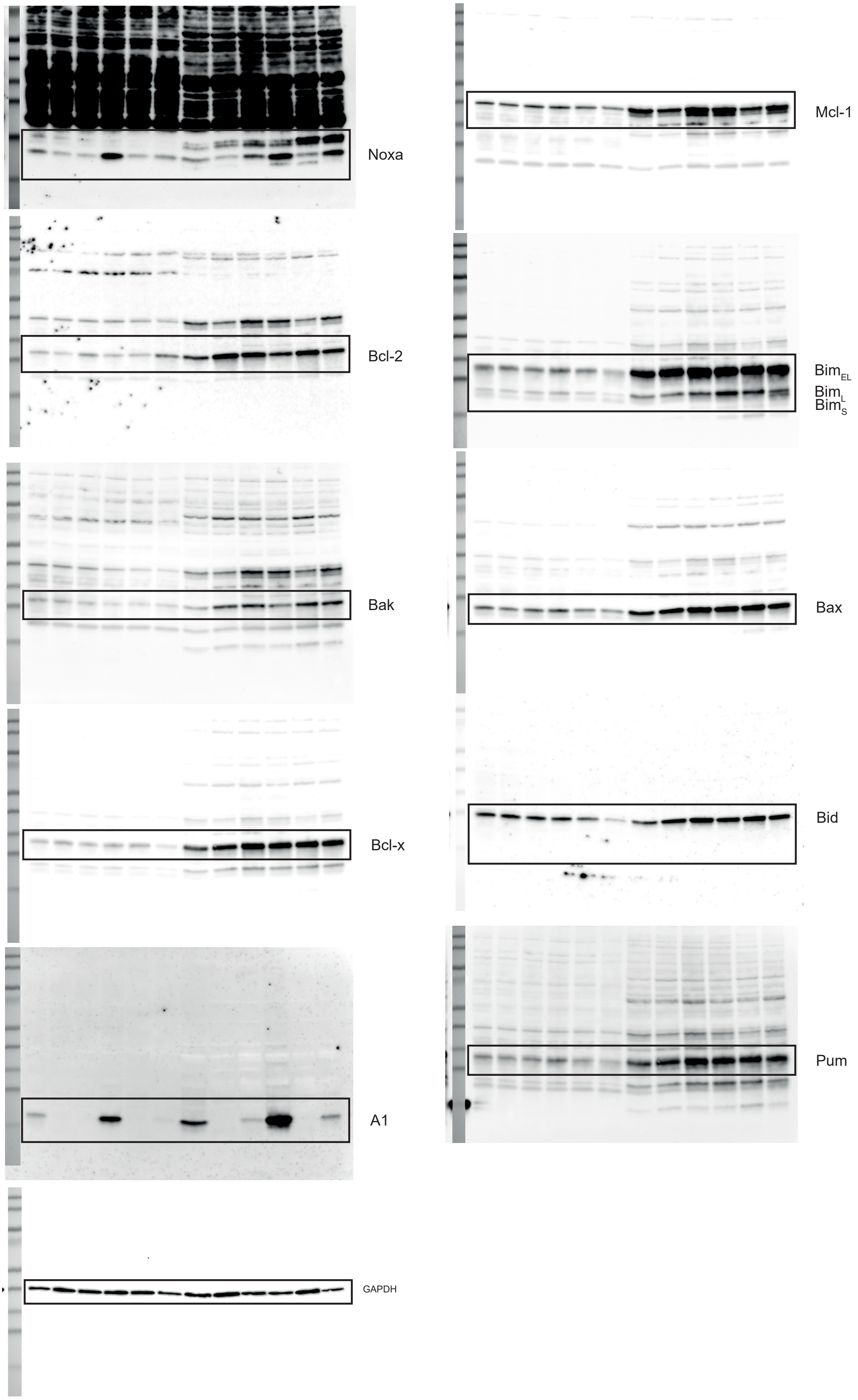

Original Western blots uncropped

related to Fig. S5b

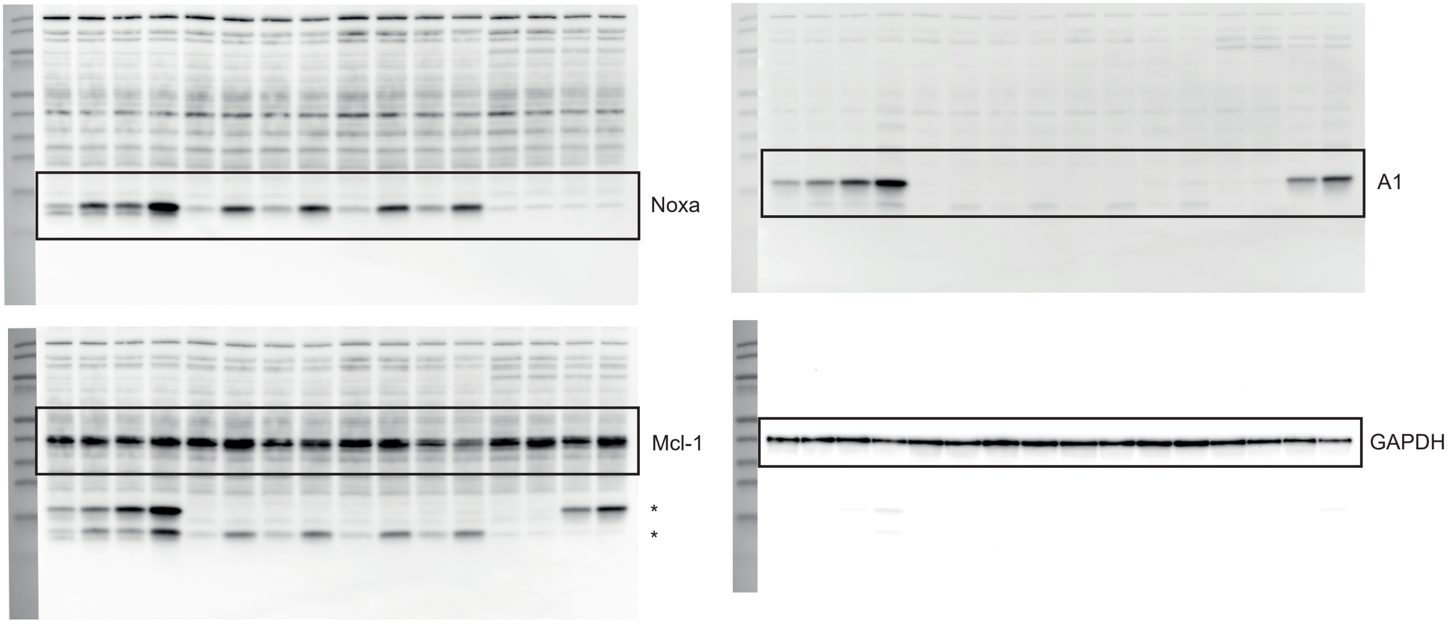

Supplement: Supplementary file 2 — Original Western Blots uncropped [file 41419_2024_7064_MOESM2_ESM.pdf]
